# Female reproductive dormancy in *Drosophila* is regulated by DH31-producing neurons projecting into the corpus allatum

**DOI:** 10.1242/dev.201186

**Published:** 2023-05-23

**Authors:** Yoshitomo Kurogi, Eisuke Imura, Yosuke Mizuno, Ryo Hoshino, Marcela Nouzova, Shigeru Matsuyama, Akira Mizoguchi, Shu Kondo, Hiromu Tanimoto, Fernando G. Noriega, Ryusuke Niwa

**Affiliations:** ^1^ Degree Programs in Life and Earth Sciences, Graduate School of Science and Technology, University of Tsukuba, Tennodai 1-1-1, Tsukuba, Ibaraki 305-8572, Japan; ^2^ Graduate School of Life and Environmental Sciences, University of Tsukuba, Tennodai 1-1-1, Tsukuba, Ibaraki 305-8572, Japan; ^3^ Life Science Center for Survival Dynamics, Tsukuba Advanced Research Alliance (TARA), University of Tsukuba, Tennodai 1-1-1, Tsukuba, Ibaraki 305-8577, Japan; ^4^ Department of Biological Sciences and BSI, Florida International University, 11200 SW 8th street, Miami, FL 33199, USA; ^5^ Institute of Parasitology, Biology Center of the Academy of Sciences of the Czech Republic, 37005, České Budějovice, Czech Republic; ^6^ Faculty of Life and Environmental Sciences, University of Tsukuba, Tennodai 1-1-1, Tsukuba, Ibaraki 305-8572, Japan; ^7^ Division of Liberal Arts and Sciences, Aichi Gakuin University, 12 Araike, Iwasaki-cho, Nisshin, Aichi 470-0195, Japan; ^8^ Department of Biological Science and Technology, Faculty of Advanced Engineering, Tokyo University of Science, Niijuku 6-3-1, Katsushika-ku, Tokyo 125-8585, Japan; ^9^ Invertebrate Genetics Laboratory, National Institute of Genetics, Yata 111, Mishima, Shizuoka 411-8540, Japan; ^10^ Graduate School of Life Sciences, Tohoku University, Katahira 2-1-1, Sendai, Miyagi 980-8577, Japan; ^11^ Department of Parasitology, University of South Bohemia, České Budějovice 37005, Czech Republic

**Keywords:** Reproductive dormancy, Diapause, Juvenile hormone, Corpus allatum, Diuretic hormone 31, *Drosophila*

## Abstract

Female insects can enter reproductive diapause, a state of suspended egg development, to conserve energy under adverse environments. In many insects, including the fruit fly, *Drosophila melanogaster*, reproductive diapause, also frequently called reproductive dormancy, is induced under low-temperature and short-day conditions by the downregulation of juvenile hormone (JH) biosynthesis in the corpus allatum (CA). In this study, we demonstrate that neuropeptide Diuretic hormone 31 (DH31) produced by brain neurons that project into the CA plays an essential role in regulating reproductive dormancy by suppressing JH biosynthesis in adult *D. melanogaster*. The CA expresses the gene encoding the DH31 receptor, which is required for DH31-triggered elevation of intracellular cAMP in the CA. Knocking down *Dh31* in these CA-projecting neurons or *DH31 receptor* in the CA suppresses the decrease of JH titer, normally observed under dormancy-inducing conditions, leading to abnormal yolk accumulation in the ovaries. Our findings provide the first molecular genetic evidence demonstrating that CA-projecting peptidergic neurons play an essential role in regulating reproductive dormancy by suppressing JH biosynthesis.

## Teaser

**Highlighted Article:** Diuretic hormone 31-producing neurons, projecting to the corpus allatum, are essential for female reproductive dormancy through the suppression of juvenile hormone in the fruit fly *Drosophila melanogaster*.

## INTRODUCTION

Unfavorable seasonal changes for prolonged durations, such as extremely low temperatures and food shortages during winter, may be challenging for the survival of animals in temperate zones. During such unfavorable conditions, organisms often suspend or retard their normal development, growth and physiological functions, a process known as diapause (Hand et al., 2016). Diapause has been intensively studied in insects as its control could benefit many aspects of industry and agriculture (Denlinger, 2008, 2022; Denlinger et al., 2012). Previous studies have revealed that a fraction of insects enter diapause at the adult stage under diapause-inducing conditions, leading to multiple metabolic and behavioral changes, including slowed or halted reproductive maturation known as reproductive diapause (Denlinger, 2022; Hutfilz, 2022; Kurogi et al., 2021). As female insect adults allocate extensive energy resources toward oogenesis (Wheeler, 2003), reproductive diapause allows them to reduce their energy consumption and then resume reproduction under more favorable conditions.

Reproductive diapause in insects is regulated by a complex interplay between multiple hormones and neurotransmitters (Denlinger, 2022; Denlinger et al., 2012; Kurogi et al., 2021). Among these, reduction in the titer of juvenile hormone (JH), an insect-specific sesquiterpenoid hormone (Qu et al., 2018; Riddiford, 2020), has been extensively studied, revealing its vital role in regulating reproductive diapause in female adult insects (Kurogi et al., 2021). As JHs are essential for promoting vitellogenesis under non-diapause-inducing conditions, a reduction in hemolymph JH levels is required for suppressing vitellogenesis, leading to reproductive diapause in females (Denlinger et al., 2012; Santos et al., 2019). In many insect species, the reduction of hemolymph JH levels correlates with the downregulation of JH biosynthesis in a specialized endocrine organ called the corpus allatum (CA) (Denlinger et al., 2012; Hand et al., 2016; Kurogi et al., 2021). Previous studies have demonstrated that surgical amputation of the nervous connection between the brain and the CA impairs the induction of reproductive diapause (Hodková, 1976; Kotaki and Yagi, 1989). These observations suggest the presence of a mechanism of signal transduction by which information about unfavorable environmental conditions is processed in the brain and transmitted to the CA to reduce JH biosynthesis.

Previous studies have demonstrated that brain neurons projecting to the CA play crucial roles in the induction of reproductive diapause in multiple insect species (Denlinger, 2002, 2022; Denlinger et al., 2012). For example, some neurons with their cell bodies in the anterior midline of the brain (pars intercerebralis; PI), project to the CA in the common ground-hopper (*Tetrix undulata*; Poras, 1982), linden bug (*Pyrrhocoris apterus*; Hodková, 1976), blowfly (*Protophormia terraenovae*; Shiga and Numata, 2000; Shiga et al., 2000) and brown-winged green bug (*Plautia stali*; Matsumoto et al., 2013). A study reported that specific PI neurons in *P. stali* produce *P. stali* myoinhibitory peptide (Plast-MIP), a neuropeptide that inhibits JH biosynthesis in CA under diapause-inducing conditions (Hasebe and Shiga, 2021b; Hasegawa et al., 2020; Matsumoto et al., 2017). It has also been demonstrated that a group of neurons, the cell bodies of which are located on the lateral side of the brain (pars lateralis; PL), project to the CA in *P. terraenovae* (Shiga and Numata, 2000), the bean bug (*Riptortus pedestris*; Shimokawa et al., 2008), the Colorado potato beetle (*Leptinotarsa decemlineata*; de Wilde and de Boer, 1969) and the grasshopper (*Locusta migratoria*; Poras et al., 1983). In all these cases, PL neurons appear to play an inhibitory role in ovarian development under diapause-inducing conditions. Moreover, cauterization experiments in *L. decemlineata* and *L. migratoria* have suggested that the PL plays an inhibitory role in JH biosynthesis under diapause-inducing conditions (Khan et al., 1986; Poras et al., 1983). In *P. terraenovae*, specific PL neurons projecting to the CA produce neuropeptides, including cholecystokinin-8 and FMRF-amide (Hamanaka et al., 2007, 2009). However, despite their crucial role in regulating reproductive diapause, the role of CA-projecting PL neurons in reproductive diapause has not been genetically confirmed. Moreover, it is unclear which neurotransmitters or neuropeptides in CA-projecting PL neurons are responsible for regulating reproductive diapause in insect species.

The fruit fly, *Drosophila melanogaster*, also undergoes the suppression of oogenesis in low-temperature and short-day conditions (Saunders et al., 1989). In the most recent pieces of literature, the reproductive diapause-like status of *D. melanogaster* is frequently called ‘reproductive dormancy’ (see Kurogi et al., 2021 for detailed discussion). Therefore, in accordance with recent studies, we will use the term ‘reproductive dormancy’ to refer to suppressing oogenesis under diapause-inducing conditions. Studies on *D. melanogaster* reproductive dormancy with powerful genetic tools have contributed substantially to the discovery of several regulatory mechanisms of insect reproductive dormancy. Several studies have confirmed that JH biosynthesis and signaling are essential for the regulation of reproductive dormancy in *D. melanogaster* (Andreatta et al., 2018; Ojima et al., 2018; Saunders et al., 1990). In this study, we report the identification and characterization of CA-projecting PL neurons that produce the neuropeptide Diuretic hormone 31 (DH31). DH31 is known to play versatile roles in insects, particularly in *D. melanogaster*, such as diuretic action, establishment of daily temperature preference rhythms, locomotor activity, sleep regulation, midgut contraction frequency, intestinal immune responses and nutrient-dependent regulation of courtship (Benguettat et al., 2018; Furuya et al., 2000; Goda et al., 2016, 2018, 2019; Head et al., 2015; Kaneko et al., 2012; Kunst et al., 2014; Lin et al., 2022; Vanderveken and O'Donnell, 2014). In this study, we propose that DH31 signaling to the CA plays an important role in the inhibition of JH biosynthesis under dormancy-inducing conditions, leading to the induction of reproductive dormancy through the inhibition of JH-mediated maturation of eggs.

## RESULTS

### A subset of DH31-producing neurons projects into the *D. melanogaster* CA

**Fig. 1.**
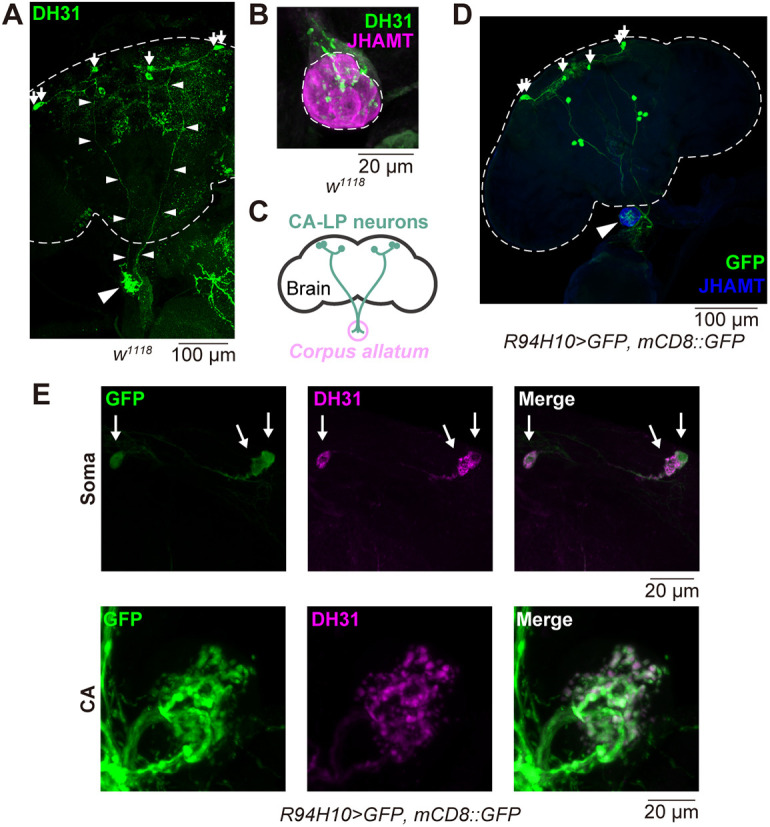
**Anatomical characterization of CA-LP neurons.** (A) Immunostaining with anti-DH31 antibody in the adult central brain (outlined by dashed lines) and the corpus allatum (CA) of wild-type (*w^1118^*) adult females. The upper and lower parts of the photograph correspond to the dorsal and ventral sides of the central brain, respectively. Axonal processes from the cell bodies to the CA (large arrowhead) are indicated by the small arrowheads. Small arrows indicate the cell bodies of CA-LP neurons. (B) Immunostaining signal in the CA of wild-type (*w^1118^*) adult females 4 days after eclosion. The anti-DH31 antibody (green) was employed along with the anti-JHAMT antibody, which was used to visualize the CA (magenta). DH31-positive puncta are observed in the CA region. Note that DH31-immunoreacive varicosities were observed inside the CA. Dashed line indicates the outline of the CA. (C) A schematic representing the anatomy of brain, CA-LP neurons and CA. (D) Transgenic visualization of CA-LP neurons by GFP driven by *R94H10-GAL4*, which specifically labels CA-LP neurons and other small subsets of neurons. Samples were immunostained with anti-GFP (green) and anti-JHAMT (blue) antibodies. The CA is marked with a large arrowhead. Dashed line indicates the outline of the brain. Small arrows indicate the cell bodies of CA-LP neurons. (E) Immunostaining signal with anti-GFP (green) and anti-DH31 (magenta) antibodies in the brain region, including the soma of CA-LP neurons (arrows), and in the CA region in an adult female *R94H10-GAL4 UAS-GFP UAS-mCD8::GFP*.

In our previous studies, we have identified several neurons projecting to various endocrine organs, including the CA of *D. melanogaster* (Imura et al., 2020; Mizuno et al., 2021; Shimada-Niwa and Niwa, 2014). To further identify CA-projecting neurons, we conducted immunostaining experiments using antibodies against different neuropeptides, and investigated whether any of these peptidergic-cell neuronal processes innervate the CA, as described previously for the hugin-positive neurons (Mizuno et al., 2021). Our results demonstrated that DH31-immunoreactive fibers and varicosities were present within the CA (Fig. 1A,B). A trace back of these CA-projecting DH31-producing neurons (Fig. 1A) led to three pairs of cell bodies located on the dorsal side of the central brain (Fig. 1A,C). Among these, two cell bodies in the brain hemisphere were clustered, whereas another cell body was located closer to the midline. We also found that the *R21C09-GAL4* driver, in which the *GAL4* transgene is expressed under the control of part of a *Dh31* enhancer, labeled these CA-projecting neurons. The *R21C09-GAL4*-driven GFP signals were observed in three pairs of the central brain neurons and neuronal processes in the CA region, which were co-immunostained with an anti-DH31 antibody (Fig. S1A,B).

A previous study identified one pair of CA-projecting lateral protocerebrum (CA-LP) 1 neurons and two pairs of CA-LP2 neurons, which innervate the CA in *D. melanogaster* larvae (Ádám et al., 2003; Siegmund and Korge, 2001). However, the neurotransmitters produced by these neurons remain unknown. As the positions of the cell bodies of the DH31-producing CA-projecting neurons were similar to those of CA-LP1 and CA-LP2 neurons, we examined whether the CA-LP1 and CA-LP2 neurons produced DH31. We found that *Kurs21-GAL4*-driven GFP, which reportedly marks CA-LP1 and CA-LP2 neurons (Fig. S1C; Siegmund and Korge, 2001), labeled the projection of the neurites to the CA in the wandering third-instar larvae (Fig. S1D) as well as in adults (Fig. S1E,F). Moreover, the cell bodies and CA-projecting neurites of CA-LP1 and CA-LP2 neurons were immunostained with the anti-DH31 antibody in both larval and adult stages (Fig. S1C-F). Therefore, the DH31-producing-CA-projecting neurons in the *D. melanogaster* adult stage are identical to the CA-LP1 and CA-LP2 neurons described in its larval stage. Hereafter, we designate these three pairs of CA-projecting DH31-producing neurons, corresponding to both CA-LP1 and CA-LP2 neurons, as ‘CA-LP neurons’.

*R21C09-GAL4* and *Kurs21-GAL4* were active in many neurons in the central brain, in addition to the CA-LP neurons (Fig. S1G,H). We thus searched for a *GAL4* driver that would be active in a restricted group of neuronal cells including the CA-LP neurons. We browsed large collections of adult fly images from the FlyLight database of the Janelia Research Campus, Howard Hugh Medical Institute (https://flweb.janelia.org/cgi-bin/flew.cgi). The FlyLight database provides large anatomical image datasets and a well-characterized collection of *GAL4* lines, allowing us to visualize individual neurons in the *D. melanogaster* central nervous system (Jenett et al., 2012). We manually checked most of the images of *GMR GAL4* lines deposited in FlyLight and found that *R94H10-GAL4* labeled CA-LP neurons and a few additional neurons (Fig. 1D,E; Fig. S1I,J). In the central brain, *R94H10-GAL4*-positive neurons other than CA-LP neurons were DH31-negative (Fig. S1I). In the ventral nerve cord (VNC), in which some neurons are known to express *Dh31* (Mandel et al., 2018), almost all *R94H10-GAL4*-positive neurons were DH31-negative, while a few cells were DH31-immunoreactives (Fig. S1J). However, as described later, we had evidence indicating that *R94H10-GAL4*- and DH31-double positive cells are not involved in reproductive dormancy. Furthermore, although *Dh31* is expressed in enteroendocrine cells (Veenstra et al., 2008), *R94H10-GAL4* was not active in these gut cells (Fig. S1K). Therefore, in this study, we used the *GAL4* driver to manipulate gene expression in CA-LP neurons.

**Fig. 2.**
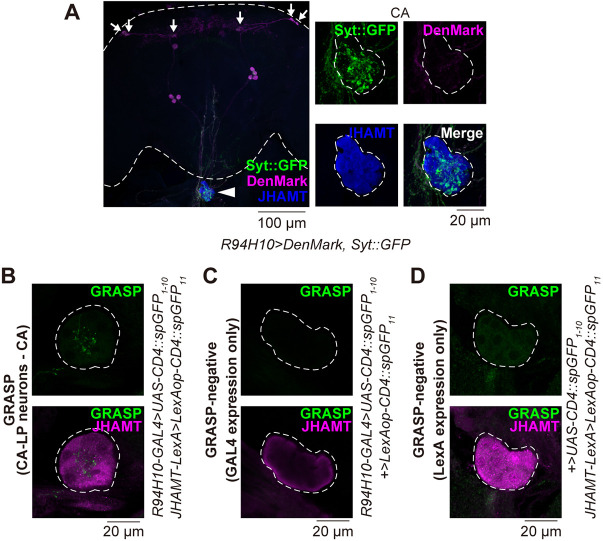
**Axonal termini of CA-LP neurons physically contact the CA.** (A) (Left) Visualization of axons and dendrites of CA-LP neurons stained by synaptotagmin-GFP (SytGFP, green) and DenMark (magenta) driven by *R94H10-GAL4*. The CA (large arrowhead) is visualized by immunostaining with anti-JHAMT (blue). Dashed line indicates the outline of the brain. (Right) Magnified view of the left panel focusing on the CA region. SytGFP, but not DenMark, signals were observed in the CA region, indicating that axons of CA-LP neurons innervate the CA. Small arrows indicate the cell bodies of CA-LP neurons. Dashed line indicates the outline of the CA. (B) The GRASP signal was employed to visualize the close physical contact between CA-LP neurons and the CA. (C,D) GFP signals of GRASP-negative control adult females. (C) GRASP signals in the CA region of a virgin female expressing *GAL4* in CA-LP neurons but not *LexA* in the CA (*+/+; R94H10-GAL4/UAS-CD4::spGFP_1-10_ LexAOP>CD4::spGFP_10_*). (D) GRASP signals in the CA region of a virgin female expressing *LexA* in the CA but not *GAL4* in CA-LP neurons (+/*JHAMT-LexA; +/UAS-CD4::spGFP_1-10_ LexAop-CD4::spGFP_10_*). Dashed line indicates the outline of the CA.

Thereafter, we examined the distribution of axonal termini and dendrites in the CA-LP neurons. *Synaptotagmin::GFP* (*Syt::GFP*) and *DenMark* transgenes, the translated products of which were localized at the axonal termini and dendrites respectively (Nicolaï et al., 2010; Zhang et al., 2002), were driven by *R94H10-GAL4*. We found that the Syt::GFP and DenMark signals were primarily observed in the CA region and in the dorsal side of the central brain respectively (Fig. 2A), suggesting that the DH31-immunoreactive fibers in the CA regions are axons. Furthermore, GFP reconstitution across synaptic partners (GRASP) analysis (Feinberg et al., 2008), in which two complementary fragments of GFP were expressed in CA-LP neurons and the CA, respectively, revealed that GRASP signals were detected inside the CA (Fig. 2B-D). These results suggest that CA-LP neurons project to the CA and are in physical contact with the CA.

### A subset of circadian clock neurons innervates the CA-LP neurons

To anatomically characterize CA-LP neurons, we browsed the publicly available connectome database of adult *D. melanogaster* brains to search for neurons that connect upstream of the CA-LP neurons (see Materials and Methods). In the available connectome data of neurons labeled with *R21C09-GAL4*, the two pairs of clustered CA-LP neurons have been annotated (Fig. S2A-D). The anatomical positions of the cell bodies and axons of these neurons suggested that they correspond to the CA-LP2 neurons (Fig. S1C,D; Siegmund and Korge, 2001). In contrast, another pair of CA-LP neurons that was similar to CA-LP1 neurons was not clearly identified in the available database. Therefore, in our connectome analysis, we focused on CA-LP2 neurons that exhibited 1780 connections with other neurons. Among these neurons, we found that several clock neurons, including the fifth sLN-v, DN1p, LNd and s-LNv, were connected to the CA-LP2 neurons (Fig. S2E). These results suggest that the circadian clock may modulate the function of the CA-LP neurons.

s-LNvs are involved in regulating reproductive dormancy in *D. melanogaster* (Nagy et al., 2019). Moreover, the s-LNvs innervate neurons located in the PL, which closely corresponds to the LP region in the brain in which the CA projecting neurons are located in several insects, including the *P. terraenovae* (Hamanaka et al., 2005; Meuti and Denlinger, 2013; Shiga and Numata, 2009)*.* Therefore, we examined the relationship between s-LNvs and CA-LP neurons in *D. melanogaster*. The morphology of s-LNvs can be visualized using an anti-pigment dispersing factor (PDF) antibody (Helfrich-Förster, 1995). We found that the axonal termini of s-LNvs were in close proximity to the neuronal processes of CA-LP neurons (Fig. S2F,G). We initially expected that the PDF receptor (Pdfr) would be present in CA-LP neurons; however, *Pdfr* knock-in *T2A GAL4* was absent in these neurons (Fig. S2H). In contrast, the short neuropeptide F (sNPF), which is known to be present in s-LNvs (Johard et al., 2009), might be involved, as the *s-NPF receptor* (*s-NPF-R*) knock-in *T2A GAL4* was positive in these neurons (Fig. S2I). These results suggest that CA-LP neurons, to an extent, have chemical connections with s-LNvs, at least through sNPF.

### The CA-LP neurons are required for inducing reproductive dormancy

Considering that CA-LP neurons are located in the lateral protocerebrum and in proximity to the axon termini of s-LNvs, the morphological and anatomical features of *D. melanogaster* CA-LP neurons are substantially similar to those of *P. terraenovae* CA-projecting neurons (Meuti and Denlinger, 2013; Shiga et al., 2000). A previous study reported that CA-projecting neurons negatively regulate the induction of reproductive dormancy in *P. terraenovae*, as surgical amputation of the axons of CA-projecting neurons caused abnormal egg production, even under dormancy-inducing conditions (Matsuo et al., 1997). Therefore, we examined whether CA-LP neurons were also involved in reproductive dormancy in *D. melanogaster*.

**Fig. 3.**
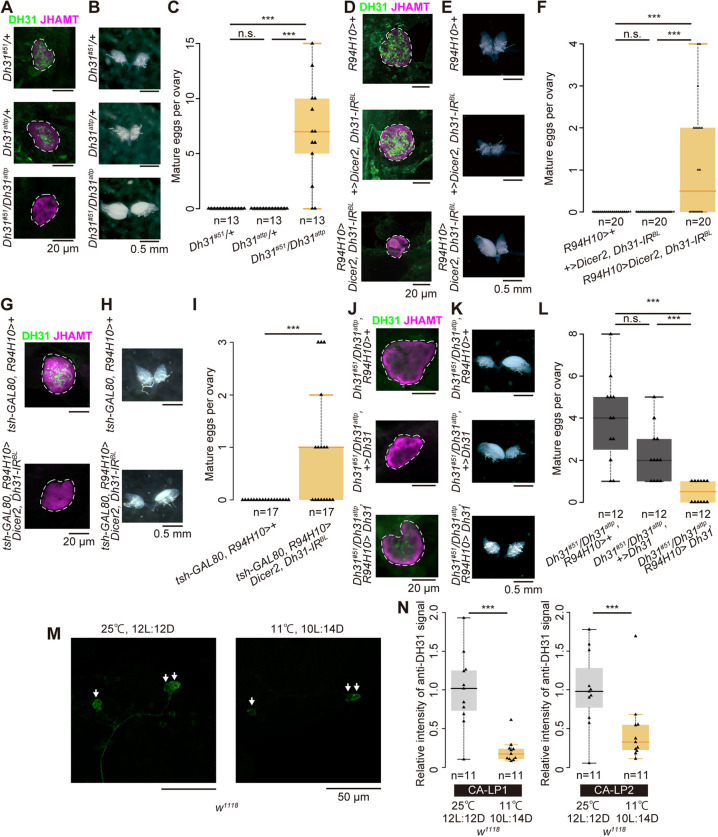
**The loss of DH31 function in CA-LP neurons fails to induce reproductive dormancy.** (A) Immunostaining signals of anti-DH31 antibody (green) in the CA region of control and *Dh31* genetic mutant females. CA was visualized with an anti-JHAMT antibody (magenta). Note that DH31 puncta were diminished in the CA region of *Dh31* trans-heterozygous mutant females. (B,C) Mature egg formation in control and *Dh31* genetic mutant females under dormancy-inducing conditions. Representative images of the ovaries (B) and quantification of mature eggs per ovary (C). (D-I) Phenotypes of *R94H10-GAL4* driven *Dh31* RNAi adult females in the absence (D-F) and presence (G-I) of *tsh-GAL80* transgene. (D,G) Immunostaining signals of the anti-DH31 antibody (green) in the CA region of control and *Dh31* RNAi adult females. CA was visualized using an anti-JHAMT antibody (magenta). DH31 puncta were diminished in the CA region of *Dh31* RNAi animals. (E,F,H,I) Mature egg formation in control and *Dh31* RNAi females under dormancy-inducing conditions. Representative images of the ovaries (E,H) and quantification of mature eggs per ovary (F,I). (J) Immunostaining signals of the anti-DH31 antibody (green) in the CA region of *Dh31* trans heterozygous mutant females in the presence or absence of CA-LP neuron-specific expression of *Dh31* cDNA under dormancy-inducing conditions. DH31 puncta were recovered in the CA region of the transgenic rescue animals. (K,L) Mature egg formation in *Dh31* trans-heterozygous mutant females with or without CA-LP neuron-specific expression under dormancy-inducing conditions. Representative images of the ovaries (K) and quantification of mature eggs per ovary (L). (M,N) Anti-DH31 immunoreactivity in the soma of CA-LP1 and CA-LP2 neurons between non-dormancy- and dormancy-inducing conditions. *w^1118^* was used for the analysis. Representative images of anti-DH31 immunostaining signals in the soma of CA-LP1 and CA-LP2 neurons (M) and quantitative comparison of anti-DH31 immunoreactivity in the soma of CA-LP1 and CA-LP2 neurons (N). Small arrows indicate the cell bodies of CA-LP neurons. ***P<0.001 (Wilcoxon rank sum test with Bonferroni's correction for C,F,I and L; unpaired two-tailed Student's *t*-test for N.). n.s., not significant. Box plots show the median values (middle bars) along with the first to third interquartile ranges (boxes). The whiskers represent 1.5 times the interquartile ranges. Samples were derived from virgin females 12 days after eclosion under dormancy-and non-dormancy-inducing conditions. Dashed line indicates the outline of the CA.

We first examined whether genetic mutants with *Dh31* loss of function displayed any phenotypes of reproductive dormancy in virgin females. We confirmed that DH31 immunoreactivity in the CA region was diminished in the *Dh31* loss-of-function flies (Fig. 3A), suggesting that DH31 is not active in the CA of mutant insects. Furthermore, under dormancy-inducing conditions (at 11±0.5°C under 10 light/14 h dark cycle), *Dh31* loss of function in females led to significant enlargement of the ovaries compared with those in control females that exhibited the typical dormancy-induced reduction of ovarian development (Fig. 3B). Finally, *Dh31* loss-of-function mutations in females led to mature egg production compared with the control females (Fig. 3C).

We next examined whether the DH31 peptide, produced in CA-LP neurons, was a crucial regulator of reproductive dormancy. For this purpose, we knocked down *Dh31* specifically in CA-LP neurons using a transgenic RNAi technique. An *R94H10-GAL4*-driven *Dh31* inverted repeat (IR) construct eliminated almost all the DH31 protein in the CA (Fig. 3D). Under dormancy-inducing conditions, RNAi-treated females displayed greater ovarian development and higher production of mature eggs than the control females (Fig. 3E,F).

We also investigated whether loss of *Dh31* function affected mature egg formation under non-dormancy-inducing conditions. However, in this case, we did not observe any difference in mature egg production between control and *Dh31* genetic mutant females (Fig. S3A) or between control and *R94H10-GAL4*-driven *Dh31* RNAi animals (Fig. S3B). These results suggest that DH31 is necessary to inhibit oogenesis during dormancy-inducing conditions, but not under non-dormancy-inducing conditions.

As we mentioned earlier, a few *R94H10-GAL4*-positive neurons in the VNC were DH31-positive (Fig. S1J; Fig. S3C). To exclude the possibility that these DH31-positive VNC neurons are involved in regulating reproductive dormancy, we used *teashirt* (*tsh*)*-GAL80* to silence GAL4 activity in the VNC (Simpson, 2016) in combination with *R94H10-GAL4* to knockdown *Dh31* using RNAi. We confirmed that the GAL4 activity in *R94H10-GAL4; tsh-GAL80* flies was efficiently silenced in the *R94H10-GAL4*- and DH31-double positive VNC neurons (Fig. S3D). Regarding the DH31 immunoreactivity in the CA and the number of mature eggs, *R94H10-GAL4; tsh-GAL80*-driven *Dh31-IR* flies exhibited almost identical phenotypes to *R94H10-GAL4*-driven *Dh31-IR* flies without *tsh-GAL80* (Fig. 3G-I). These results suggest that DH31-positive VNC neurons are not involved in regulating reproductive dormancy.

We implemented an additional genetic approach, in which a *Dh31* transgene was driven specifically in CA-LP neurons in the genetic background of the *Dh31* loss-of-function mutation. An *R94H10-GAL4*-driven *Dh31* overexpression restored DH31 protein levels in the CA of the mutant females (Fig. 3J). Although loss of DH31 function in females failed to induce reproductive dormancy, *Dh31* overexpression rescued the phenotypes of reproductive dormancy (Fig. 3K,L).

Lastly, we examined the sufficiency of CA-LP neurons to suppress oogenesis. For this purpose, we overexpressed *TrpA1*, a temperature-sensitive cation channel gene (Hamada et al., 2008), in CA-LP neurons under non-dormancy-inducing conditions. Both the control and *TrpA1*-overexpressing virgin females at permissive temperature (21°C) had the normal number of mature eggs. On the other hand, at the restrictive temperature (29°C), the *TrpA1*-overexpressing virgin females had less mature eggs compared with controls (Fig. S3E), suggesting that CA-LP neuronal activity is sufficient to suppress oogenesis. Taken together, these results support the idea that DH31 produced by CA-LP neurons plays an essential role in the suppression of oogenesis under dormancy-inducing conditions.

### Comparison of *Dh31* expression, DH31 protein levels and CA-LP neuronal activity between dormancy- and non-dormancy-inducing conditions

To understand whether and how CA-LP neurons are regulated in response to dormancy-inducing conditions, we compared *Dh31* expression and DH31 protein levels between dormancy- and non-dormancy-inducing conditions. Levels of *Dh31* expression and DH31 protein in CA-LP neurons were confirmed by the *Dh31* enhancer (*R21C09*)-*GAL4* and by immunostaining with an anti-DH31 antibody, respectively. We found that *Dh31* expression levels were upregulated in CA-LP1 neurons but remained unchanged in CA-LP2 neurons under dormancy-inducing conditions (Fig. S4A). In contrast, DH31 protein levels were notably reduced in both CA-LP1 and CA-LP2 neurons under dormancy-inducing conditions (Fig. 3M,N). These results might reflect enhancement of *Dh31* transcription, at least in CA-LP1 neurons, and DH31 release from CA-LP neurons in response to dormancy-inducing conditions. To further investigate whether dormancy-inducing conditions affect the function of CA-LP neurons, we conducted a transcriptional reporter of intracellular Ca^2+^ (TRIC) assay, which is used to assess neuronal activities (Gao et al., 2015). However, we found no obvious elevation of TRIC signals, corresponding to intracellular Ca^2+^ levels, in CA-LP neurons under dormancy-inducing conditions (Fig. S4B,C). Therefore, although we have demonstrated that CA-LP neurons are regulated in response to dormancy-inducing conditions, the exact mechanism of this regulation is still unclear.

### The DH31 receptor in the CA is required for the induction of reproductive dormancy

**Fig. 4.**
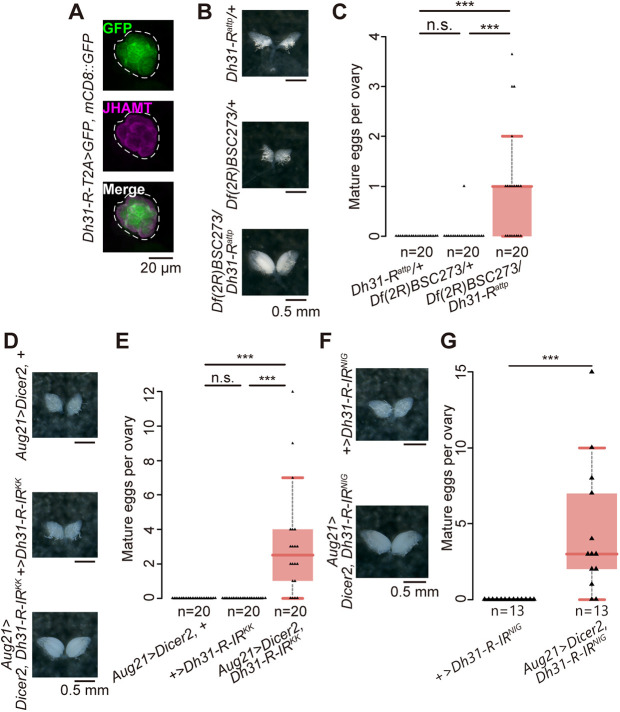
**The loss of DH31 receptor function in the CA leads to failure in the induction of reproductive dormancy.** (A) Expression of *Dh31-R* visualized using *Dh31-R-T2A-GAL4*-driven GFP (green). CA was revealed using an anti-JHAMT antibody (magenta). Dashed line indicates the outline of the CA. (B,C) Mature egg formation in control and *Dh31-R* mutant females under dormancy-inducing conditions. Representative images of the ovaries (B) and quantification of mature eggs per ovary (C). (D,E) Mature egg formation in the control and CA-specific *Dh31-R* RNAi females under dormancy-inducing conditions. Representative images of the ovaries (D) and quantification of mature eggs per ovary (E). Note that these data were obtained from the Vienna *Drosophila* Resource Center KK RNAi strain. (F,G) Mature egg formation in the control and CA-specific *Dh31-R* RNAi females under dormancy-inducing conditions. These data were obtained from the transgenic RNAi strain from the National Institute of Genetics. Representative images of the ovaries (F) and quantification of mature eggs per ovary (G). ***P<0.001 (Wilcoxon rank-sum test with Bonferroni correction for C,E,G). n.s., not significant. Box plots show the median values (middle bars) along with the first to third interquartile ranges (boxes). The whiskers represent 1.5 times the interquartile ranges. Samples were derived from virgin females 12 d after their transfer to dormancy-inducing conditions.

As CA-LP neurons project to and are in close contact with the CA, we examined whether a specific receptor for DH31 (DH31-R) (Johnson et al., 2005) was present in the CA. For this purpose, we generated a knock-in strain by inserting the *T2A-GAL4* cassette into the *Dh31-R* locus (49). This transgenic line was used to confirm *Dh31-R* expression in the CA (Fig. 4A), and the expression levels of *Dh31-R* in the CA were not different between dormancy- and non-dormancy-inducing conditions (Fig. S5A). We then conducted phenotypic analyses of the genetic mutants that presented a loss of DH31-R function. Under dormancy-inducing conditions, *Dh31-R* mutant females produced more mature eggs compared with controls (Fig. 4B,C). We also took an approach using transgenic RNAi to knock down *Dh31-R* in the CA. Forced expression of two independent *Dh31-R* IR constructs resulted in ∼60% and 80% reduction of *Dh31-R* mRNA levels, respectively (Fig. S5B). We observed reproductive phenotypes in females in which *Dh31-R* was knocked down in the CA with *Aug21-GAL4*, known to be active in the CA (Ádám et al., 2003; Siegmund and Korge, 2001) (Fig. 4D,E). *Aug21-GAL4*-driven RNAi phenotypes were observed using two independent *Dh31-R* RNAi lines (Fig. 4F,G). In addition, *JH acid O-methyltransferase* (*JHAMT*)*-GAL4*-driven *Dh31-R* RNAi also exhibited reproductive phenotype under dormancy-inducing conditions (Fig. S5C). These results confirm that DH31-R in the CA is required for the induction of reproductive dormancy.

Recent studies have revealed the essential role of the insulin-producing neurons (IPCs) in regulating reproductive dormancy (Kubrak et al., 2014; Kurogi et al., 2021; Nagy et al., 2019; Ojima et al., 2018). We therefore wondered whether DH31-R was also present and played an essential role in the IPCs. However, *Dh31-R-T2A-GAL4*-driven GFP signal was not observed in the IPCs marked by the presence of *Drosophila* insulin-like peptide 2 (DILP2; ILP2) (Fig. S5D). In addition, when we knocked down *Dh31-R* using *Dilp2-GAL4*-driven transgenic RNAi, mature egg production was not enhanced in the RNAi animals compared with control under dormancy-inducing conditions (Fig. S5E,F). These results suggest that DH31-R acts in the CA rather than in the IPCs to regulate reproductive dormancy.

We also examined whether mature egg formation was impaired by loss of *Dh31-R* function under non-dormancy-inducing conditions. However, under non-dormancy-inducing conditions, either *Dh31-R* genetic mutant females or CA-specific *Dh31-R* RNAi females produced a number of mature eggs comparable with control animals (Fig. S5G-I). Therefore, these results suggest that DH31-R is required for suppressing oogenesis under dormancy-, but not in non-dormancy-inducing conditions.

### DH31 induces intracellular cAMP elevation in the CA through the DH31 receptor

**Fig. 5.**
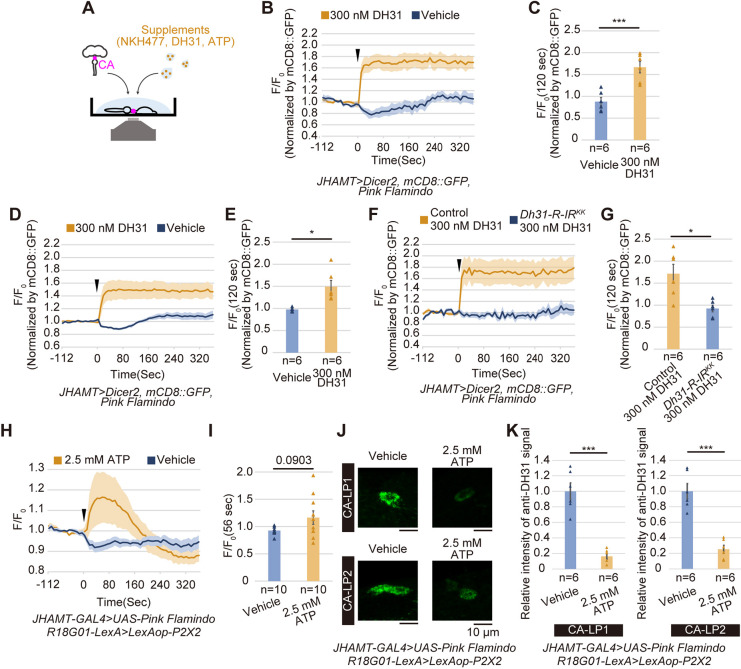
**Synthetic DH31 peptide elevates intracellular cAMP level in the CA through the DH31 receptor.** (A) A schematic showing *ex vivo* cAMP imaging. The dissected tissues, containing the brain, gut and CA, were incubated in Schneider's *Drosophila* medium in the presence or absence of synthetic DH31 peptide, ATP or NKH477. (B-E) Pink Flamindo imaging in the CA, dissected out from non-dormant (B,C) and dormant (D,E) adult females, with or without DH31 peptide administration. (B,D) Changes in the relative fluorescence intensity of *JHAMT-GAL4*-driven Pink Flamindo in the CA with or without stimulation using 0.3 μM DH31 peptide (each *n*=6). *F/F_0_* values are normalized by the signal intensity of *JHAMT-GAL4*-driven mCD8::GFP. (C,E) Quantification of normalized Pink Flamindo signals in the CA at 120 s after the stimulation. (F,G) Pink Flamindo imaging in the CA, dissected out from control and *Dh31-R* RNAi non-dormant adult females, with or without DH31 peptide administration. (F) Changes in the relative fluorescence intensity of *JHAMT-GAL4*-driven Pink Flamindo in the CA of control and *Dh31-R* RNAi females with stimulation using 0.3 μM DH31 peptide (each *n*=6). *F/F_0_* values are normalized by the signal intensity of *JHAMT-GAL4*-driven mCD8::GFP. (G) Quantification of normalized Pink Flamindo signals in the CA at 120 s after the stimulation. (H,I) Pink Flamindo imaging in the CA, dissected out from dormant flies expressing *P2X2* in CA-LP neurons, with or without ATP administration. (H) Changes in the relative fluorescence intensity of *JHAMT-GAL4*-driven Pink Flamindo in the CA of *R18G01-LexA LexAOP-P2X2* females with stimulation using 2.5 mM ATP (each *n*=10). (I) Quantification of normalized Pink Flamindo signals in the CA at 56 s after the stimulation. (J,K) Anti-DH31 signal in the CA-LP neurons in the vehicle- and ATP-treated brain-CA complexes collected after Pink Flamindo imaging shown in H and I. (J) Representative images of anti-DH31 immunostaining signals in the soma of CA-LP1 and CA-LP2 neurons. (K) Quantitative comparison of anti-DH31 immunoreactivity in the soma of CA-LP1 and CA-LP2 neurons. Data are mean±s.e.m. *P<0.05, ***P<0.001 (unpaired two-tailed Student's *t*-test for C,E,G,I and K). n.s., not significant.

We employed live imaging techniques to further understand the role of DH31-R in transmitting DH31 signals to CA cells. Considering that DH31-R is coupled to cAMP as a secondary messenger (Johnson et al., 2005), we monitored the increase in intracellular cAMP levels using a CA *ex vivo* culture system that assays the activity of the cAMP sensor probe, Pink Flamindo (Harada et al., 2017). Fly tissues containing the CA expressing the *Pink Flamindo* transgene were dissected and treated with collagenase because the CA is surrounded by a thick layer of collagens (Fig. S6A). The collagenase-treated tissue was placed in a glass-bottomed dish filled with culture medium (Fig. 5A; Fig. S6B); subsequently, the Pink Flamindo signals were recorded in the presence and absence of extracellular chemical stimuli. We first confirmed that the Pink Flamindo signals reflected intracellular cAMP levels, as the administration of the adenylyl cyclase activator NKH477 elevated Pink Flamindo signals in the CA (Fig. S6C,D). Using this *ex vivo* system, we found that the administration of the synthetic DH31 peptide resulted in the prompt elevation of Pink Flamindo signals in the CA of both non-dormant flies (Fig. 5B,C) and dormant flies (Fig. 5D,E). Importantly, the DH31-stimulated elevation of Pink Flamindo signals was not observed in flies with the *Dh31-R* knocked down in the CA (Fig. 5F,G). These results indicate that DH31 signals, via DH31-R, lead to an increase in intracellular cAMP levels in CA cells.

We also took another approach using a heterologously-expressed P2X2 purinoreceptor, which can activate neurons by bath application of ATP (Yao et al., 2012), to investigate whether the synaptic transmission of DH31 to the CA increases intracellular cAMP in the CA. In this case, we prepared adult females expressing *P2X2* driven by *R18G01-LexA*, which labeled CA-LP neurons (Fig. S6E), along with *JHAMT-GAL4*-driven *Pink Flamindo*. After the brain-CA complexes were dissected out with their intact neuronal connections, we applied ATP to the medium, which should stimulate CA-LP neurons and Pink Flamindo signals in the CA. We observed a substantial, transient elevation of Pink Flamindo signals in the CA after ATP administration compared with control vehicle administration (Fig. 5H,I), although the difference between the groups did not reach statistical significance (Fig. 5I). We should note that the preparation of the brain-CA complex was technically challenging, at least in our hands. We also found that, in the brain-complexes after Pink Flamindo imaging, DH31 protein levels in CA-LP neurons were reduced in the ATP-treated samples compared with the vehicle-treated ones (Fig. 5J,K). This observation is consistent with our data showing that DH31 protein levels in CA-LP neurons were reduced in dormancy-inducing conditions (Fig. 3M,N). Therefore, our data suggest, to some extent, the involvement of the synaptic DH31 transmission in the elevation of intracellular cAMP in the CA.

Based on the results above, we hypothesized that the increased intracellular cAMP in the CA should lead to a suppression of JH biosynthesis, inducing reproductive dormancy. To test the hypothesis, we used a dominant negative form of protein kinase A (PKA^DN^), which is the major effector protein downstream of cAMP. Consistent with the fact that a forced expression of *PKA^DN^* in the CA leads to enhancing JH signaling (Andreatta et al., 2018), *JHAMT-GAL4*-driven *PKA^DN^* expression resulted in producing more mature eggs under dormancy-inducing conditions (Fig. S7A,B). These results, along with those of a previous study (Andreatta et al., 2018), suggest that the increased level of intracellular cAMP in the CA negatively regulates oogenesis via the suppression of JH biosynthesis.

### DH31 signaling in the CA plays a role in decreasing hemolymph JH titers

**Fig. 6.**
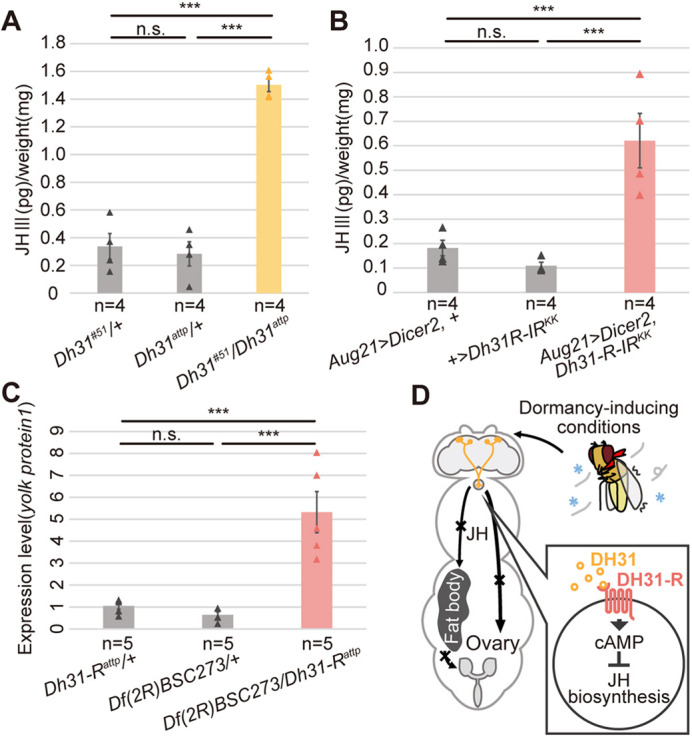
**Hemolymph JH III titers are increased after the loss of DH31 signaling.** (A) Hemolymph JH III titers in control and *Dh31* genetic mutant females under dormancy-inducing conditions. JH III amounts are normalized by body weight. (B) Hemolymph JH III titers in control and CA-specific *Dh31-R* RNAi females under dormancy inducing conditions. JH III amounts are normalized by body weight. (C) Quantification of *yolk protein 1* mRNA in control and *Dh31-R* genetic mutant females under dormancy-inducing conditions by RT-qPCR. (D) A proposed model of DH31-dependent regulation of reproductive dormancy in *D. melanogaster*. Data are mean±s.e.m. ***P<0.001 (Tukey–Kramer's HSD test for G and H). n.s., not significant. Samples were derived from virgin females 6 days after their transfer to dormancy-inducing conditions.

The induction of *D. melanogaster* reproductive dormancy is associated with the suppression of JH biosynthesis in the CA (Andreatta et al., 2018; Ojima et al., 2018; Saunders, 1990; Tatar and Yin, 2001; Tatar et al., 2001); therefore, we examined whether DH31 signaling in the CA influenced JH titers in the hemolymph. We used liquid chromatography coupled with electrospray tandem mass spectrometry (LC-MS/MS) (Ramirez et al., 2020) to measure hemolymph titers of JH III, a major form of JH in *D. melanogaster* adult females (Bownes and Rembold, 1987; Reiff et al., 2015; Sliter et al., 1987; Sugime et al., 2017). Females with *Dh31* or *Dh31-R* loss of function exhibited a higher hemolymph JH III titer than the control females (Fig. 6A,B). In addition, under dormancy-inducing conditions, administration of the JH analog, methoprene, induced egg maturation, which phenocopied females with *Dh31* loss of function (Fig. S7C,D).

JH stimulates yolk protein synthesis in the fat body to promote mature egg production (Bownes, 1989; Flatt et al., 2005; Postlethwait and Weiser, 1973). Consistently, the mRNA levels of *yolk protein 1*, *yolk protein 2* and *yolk protein 3* were upregulated in females with loss of *Dh31-R* function (Fig. 6C; Fig. S7E,F). Altogether, these results suggest that DH31 signaling in the CA plays a crucial role in suppressing the hemolymph JH titer under dormancy-inducing conditions, leading to reproductive dormancy through the inhibition of JH-mediated oocyte maturation (Fig. 6D).

## DISCUSSION

CA-projecting PL neurons are essential regulators of reproductive dormancy in insects (de Wilde and de Boer, 1969; Poras et al., 1983; Shiga and Numata, 2000; Shimokawa et al., 2008). In addition, CA-projecting PL neurons are known to play a crucial inhibitory role in JH biosynthesis (Khan et al., 1986; Poras et al., 1983). The current study provides the first genetic evidence of the role of CA-projecting PL neurons in JH-mediated reproductive dormancy. Our results suggest that the CA-LP neuron-CA axis modulates JH biosynthesis in response to dormancy-inducing conditions, revealing a regulatory neuroendocrine mechanism underlying reproductive dormancy.

Our study confirmed that the DH31-producing CA-projecting neurons are identical to CA-LP neurons, which have been anatomically characterized in *D. melanogaster* larvae (Siegmund and Korge, 2001). Previous studies have indicated that CA-LP neurons have a negative effect on JH biosynthesis during pupal development, permitting a normal male genital rotation (Ádám et al., 2003), consistent with the inhibitory role of CA-LP neurons in JH biosynthesis during the adult stage. Nevertheless, we found that there was no phenotype of male genitalia rotation in genetic mutant males exhibiting either loss of *Dh31* or *Dh31-R* functions (Fig. S8A,B). These results indicate that, at least in the larval and/or pupal stages, CA-LP neurons might produce an additional neuropeptide or neurotransmitter, different from DH31, which also has a negative impact on JH biosynthesis in the CA. Therefore, a complete list of the neuropeptides/neurotransmitters produced in CA-LP neurons is required.

Consistent with our observations, a recent study reported that DH31 and DH31-R were not involved in *D. melanogaster* oogenesis under non-dormancy-inducing conditions (Ma et al., 2020). In addition, our data indicated that *Dh31* transcription, at least in CA-LP1 neurons, is elevated, and DH31 protein level in the soma of CA-LP neurons is reduced in response to dormancy-inducing conditions. Based on these results, we hypothesize that dormancy-inducing conditions stimulate CA-LP neurons to produce *Dh31* and release its products to the CA, which may underlie the induction of reproductive dormancy. However, it should be noted that this hypothesis requires further demonstration. In addition, another issue is that we did not observe differences in TRIC signals in CA-LP neurons between dormancy- and non-dormancy-inducing conditions. In future studies, it would be interesting to use additional neurobiological technologies besides TRIC assay to examine whether and how CA-LP neuronal activities are regulated in response to dormancy-inducing conditions. Previous research has reported that the circadian clock system is involved in the regulation of reproductive dormancy in many insect species (Denlinger, 2022; Hasebe and Shiga, 2021a; Meuti and Denlinger, 2013; Saunders, 2020), including *D. melanogaster* (Abrieux et al., 2020; Nagy et al., 2019; Saunders, 1990; Saunders et al., 1989). Therefore, we propose that, to an extent, circadian clock neurons, such as fifth s-LNv, s-LNv, LNd and DN1p are connected with CA-LP neurons and modulate their activity under dormancy-inducing conditions. In addition, sNPF secreted from s-LNv may stimulate the sNPF-R to modulate the activity of CA-LP neurons. Remarkably, a recent study showed that sNPF secreted from s-LNv cells positively regulates IPCs, leading to the suppression of reproductive dormancy in *D. melanogaster* (Nagy et al., 2019). Therefore, an alternative hypothesis is that sNPF from s-LNv simultaneously regulates CA-LP neurons and IPCs to control reproductive dormancy. If sNPF plays a suppressive role in reproductive dormancy, it should negatively regulate CA-LP neurons. Notably, an inhibitory effect of sNPF on central neurons has been reported in *D. melanogaster* (Vecsey et al., 2014).

Our studies, for the first time, identified DH31 as a neurotransmitter derived from neurons that directly innervate the CA. In contrast, recent studies using *D. melanogaster* have revealed a number of circulating factors that are crucial for regulating reproductive dormancy (Kurogi et al., 2021). Most of these studies have repeatedly emphasized the importance of insulin-like peptides (ILPs) produced in IPCs located in PI. ILPs directly stimulate CA to enhance JH biosynthesis through the PI3-kinase-mTOR pathway under non-dormancy-inducing conditions (Ojima et al., 2018). Conversely, under dormancy-inducing conditions, the production and secretion of ILPs from IPCs are inhibited, resulting in the suppression of JH biosynthesis, thus leading to reproductive dormancy (Kurogi et al., 2021). The modulation of IPC activity is mediated by multiple neuropeptides and neurotransmitters, including PDF and sNPF produced by the s-LNv cells, as well as monoaminergic neurotransmitters, such as serotonin and dopamine (Andreatta et al., 2018; Nagy et al., 2019). Dopamine also suppresses JH biosynthesis via a dopamine receptor subtype in the CA (Andreatta et al., 2018). On the other hand, the release of allatostatin C (AstC) from DN3 neurons is essential for cold-induced reproductive dormancy through the activation of cholinergic AstC receptor type 2 in clock neurons (Meiselman et al., 2022). CA-LP neurons might cooperate with these humoral factors for the proper regulation of reproductive dormancy *in vivo*; however, it is not known how all these signals are integrated to regulate reproductive dormancy.

A previous study has reported that cAMP-dependent protein kinase (PKA) negatively regulates JH biosynthesis (Andreatta et al., 2018). Considering this and our data from Pink Flamindo imaging studies, it is plausible that DH31 regulates JH biosynthesis by modulating intracellular cAMP levels; however, additional studies are required to elucidate the molecular basis of cAMP and PKA modulation of JH biosynthesis. Based on our immunostaining data, DH31 signaling did not seem to affect the protein levels of JHAMT, a crucial enzyme in the JH biosynthesis pathway (Niwa et al., 2008; Shinoda and Itoyama, 2003).

JH plays key roles in regulating numerous *D. melanogaster* adult female physiological processes, including vitellogenesis in non-dormancy-inducing conditions, ovulation, egg shape maintenance, gut remodeling, innate immunity, sleep regulation and aging (Flatt et al., 2008; Luo et al., 2021; Postlethwait and Weiser, 1973; Reiff et al., 2015; Schwenke and Lazzaro, 2017; Wu et al., 2018; Yamamoto et al., 2013). In addition, JH signaling is also essential for regulating adult male physiological processes in *D. melanogaster*, including protein synthesis in accessory glands, sleep regulation, courtship motivation and courtship-associated memory retention (Lee et al., 2017; Wijesekera et al., 2016; Wu et al., 2018; Yamamoto et al., 1988; Zhang et al., 2021). In fact, we found that CA-LP neurons are also present in adult males (Fig. S8C) and therefore it is possible that CA-LP neurons might also regulate male adult JH biosynthesis. Further studies could reveal additional roles of CA-LP neurons in modulating JH-dependent events in adult male and female flies.

Considering that DH31 is a well-conserved molecule in invertebrates (Cai et al., 2018), future studies could also investigate the role of CA-LP neurons in modulating JH-mediated reproductive dormancy in other insects. In particular, the morphology of *D. melanogaster* CA-LP neurons closely resembled that of the CA-projecting PL neurons in *P. terraenovae* (Shiga and Numata, 2000), suggesting that CA-LP neurons might be involved in the regulation of reproductive dormancy in other dipteran species.

Our study provides conclusive genetic evidence that *D. melanogaster* female reproductive dormancy is regulated by DH31-producing neurons projecting to the CA*.* Further functional analyses of the clock neurons–CA-LP neurons–CA axis in other insects could shed light on the conserved molecular mechanisms underlying the environmental adaptation of insects, including agricultural pests and infectious disease vectors. For example, a previous study has shown that DH31-positive neuronal processes innervate the CA in the cabbage root fly (*Delia radicum*), a serious pest for many Brassicaceae crops (Zoephel et al., 2012). These studies would provide important information that could help establish the foundation for the development of new approaches for the control of harmful insects. Lastly, as DH31 belongs to an evolutionarily conserved peptide family that includes mammalian calcitonin gene-related peptides (Coast et al., 2001), it would be intriguing to examine whether these related peptides are also involved in regulating dormancy or hibernation in other animal species.

## MATERIALS AND METHODS

### *Drosophila* strains and maintenance

*D. melanogaster* flies were raised until eclosion on a standard yeast-cornmeal-glucose fly medium [0.275 g agar, 5.0 g glucose, 4.5 g cornmeal, 2.0 g yeast extract, 150 μl propionic acid and 175 μl 10% butyl p-hydroxybenzoate (in 70% ethanol) in 50 ml water] at 25°C under a 12 light/12 h dark cycle. To induce reproductive dormancy, adult virgin females were collected within 6 h of eclosion and reared on standard medium at 11±0.5°C under short-day length (SD: 10 h light/14 h dark cycle) conditions for 12 days, as described in previous studies (Andreatta et al., 2018; Ojima et al., 2018). To analyze the effects of JH analogs on reproductive dormancy, virgin female flies were collected within 6 h of eclosion and reared on standard medium supplemented with 1.5 mM methoprene (Sigma-Aldrich, PESTANAL 33375, racemic mixture; 1.5 M stock was prepared in ethanol) or 0.1% ethanol (control).

The following *D. melanogaster* genetic mutant strains were used: *Dh31^#51^* (from Fumika N. Hamada, Cincinnati Children's Hospital Medical Center, USA), *Dh31^attp^* [Bloomington *Drosophila* Stock Center (BDSC), #84490] and *Dh31-R^attp^* (BDSC, #84491) (from Yi Rao, Peking University School of Life Sciences, China). The *Dh31^#51^* allele has a 730-bp deletion containing the second and third exons of the *Dh31* gene (Head et al., 2015). *Dh31^attp^* is another allele in which a 1500-bp genomic region containing all exons of the Dh31 gene was replaced with a knock-in cassette (Deng et al., 2019).

In addition, the following transgenic *D. melanogaster* strains were used: *Actin5C-GAL4* (BDSC, #3954), *Aug21-GAL4* (Siegmund and Korge, 2001) (BDSC, #30137), *Df(2R)BSC273* (BDSC, #23169), *Dilp2-GAL4* (BDSC, #37516), *Dh31-R-T2A-GAL4* (Kondo et al., 2020), *JHAMT-GAL4* (from Brigitte Dauwalder, University of Houston, USA; Wijesekera et al., 2016), *JHAMT-LexA* (from Naoki Yamanaka, U.C. Riverside, USA), *Kurs21-GAL4* (from Stéphane Noselli, Université Côte D'Azur, France; Siegmund and Korge, 2001), *LexAop-CD4::spGFP_11_,UAS-CD4::spGFP_1-10_* (BDSC, #58755), *LexAop-mCD8::GFP* (BDSC, #32203), *LexAop-P2X2* (BDSC, #76030), *Pdfr-T2A-GAL4* (Kondo et al., 2020), *R18G01-LexA* (BDSC, #52531), *R21C09-GAL4* (BDSC, #48936), *R94H10-GAL4* (BDSC, #47268), *sNPF-R-T2A-GAL4* (BDSC, #84691), *tsh-GAL80* (Simpson, 2016), *UAS-DenMark,UAS-Syt1::GFP* (BDSC, #33064), *UAS-Dh31* (FlyORF, #F003632), *UAS-Dh31-IR* (BDSC, #41957), *UAS-Dh31-R-IR^NIG^* (NIG-FLY, #17043R-1), *UAS-Dh31-R-IR^KK^* (Vienna *Drosophila* Resource Center, #101995), *UAS-Dicer2* (BDSC, #24651), *UAS-mCD::GFP* (BDSC, #32219), *UAS-mCD8::RFP, LexAop2-mCD8::GFP;nSyb-MKII::nlsLexADBDo;UAS-p65AD::CaM* (for TRIC assay; BDSC, #61679), *UAS-Pink Flamindo* (this study), *UAS-PKA^DN^* (BDSC, #35550), *UAS-TrpA1;UAS-TrpA1* (the combined strain of BDSC #26263 and #26264, carrying *TrpA1* transgenes on both the second and third chromosome), *Viking-GFP* (*Drosophila* Genomics Resource Center, #110692), and *UAS-GFP,mCD8::GFP* (from Kei Ito, University of Cologne, Germany; Ito et al., 1998). Heterozygous controls were obtained by crossing *w^1118^* with strains of genetic mutants, *GAL4* drivers or UAS effectors.

### Generation of mouse anti-DH31 antibody

Peptides corresponding to the C-terminal 16 amino acid sequence (NH_2_-AKHLMGLAAANFAGGP-NH_2_) of *Bombyx mori* DH31 (GenBank accession number BAG49567.1), with an N-terminal addition of cysteine, were synthesized and conjugated with maleimide-activated bovine serum albumin (BSA) (Imject Maleimide-Activated BSA, Thermo Fisher Scientific, 77115). The BSA-conjugated DH31 partial peptides were dialyzed with phosphate buffer saline (PBS). Subsequently, 50 μl of 1 mg/ml dialyzed conjugates was mixed with 25 μl of ABISCO-100 adjuvant (Isconova) and 25 μl of PBS. The mixture was subcutaneously injected twice into the mice and whole blood was collected 12 days after the last immunization. Blood serum was heat-inactivated at 56°C for 30 min, followed by the addition of an equal volume of saturated ammonium sulfate solution to precipitate the proteins. The precipitate was dissolved and dialyzed twice in PBS. We would like to emphasize that there was only one amino acid difference in the C-terminal 16 amino acid sequence of DH31 between *B. mori* and *D. melanogaster* (NH_2_-AKHRMGLAAANFAGGP-NH_2_; GenBank accession number NP_523514.1); therefore, cross-reactivity was expected. The anti-Dh31 antibody signal in the CA disappeared in the complete deletion mutant of Dh31 (Fig. 3A); conversely, the signal reappeared in the Dh31-overexpressing animals on the Dh31 genetic mutant background (Fig. 3J), confirming the specificity of the antibody.

### Immunohistochemistry

The tissues were dissected in PBS and fixed in 4% paraformaldehyde in PBS for 30-60 min at 25-27°C. The fixed samples were rinsed thrice in PBS, washed for 15 min with PBS containing 0.3% Triton X-100 (PBT), and treated with a blocking solution (2% BSA in PBT; Sigma-Aldrich, #A9647) for 1 h at 25-27°C or overnight at 4°C. The samples were incubated with a primary antibody in blocking solution overnight at 4°C. The primary antibodies used were as follows: chicken anti-GFP antibody (Abcam, #ab13970, 1:2000), rabbit anti-RFP antibody (Medical & Biological Laboratories, PM005, 1:2000), guinea pig anti-JHAMT antibody (Mizuno et al., 2021; 1:1000-2000); rabbit anti-JHAMT antibody (Niwa et al., 2008, 1:1000), mouse anti-DH31 antibody (this study; 1:200), guinea pig anti-DH31 (from Michael Nitabach, Yale University, USA; Kunst et al., 2014; 1:500), guinea pig anti-DILP2 (from Takashi Nishimura, Gunma University, Japan; Okamoto and Nishimura, 2015; 1:200) and rabbit antibody against PDF of the cricket *Gryllus bimaculatus* (from Outa Uryu and Kenji Tomioka, Okayama University, Japan; Abdelsalam et al., 2008; 1:2000). Notably, previous studies have confirmed that anti-cricket PDF antibodies cross-react with the *D. melanogaster* PDF protein (Miyasako et al., 2007; Umezaki et al., 2012). The samples were rinsed thrice with PBS and then washed for 15 min with PBT, followed by incubation with fluorophore (Alexa Fluor 488, 546, or 633)-conjugated secondary antibodies (Thermo Fisher Scientific; 1:200) in blocking solution for 2 h at room temperature or overnight at 4°C. The secondary antibodies (Thermo Fisher Scientific; 1:200) used were as follows: goat anti-chicken secondary antibody Alexa Fluor 488(A32931; RRID: AB_2762843), goat anti-mouse secondary antibody Alexa Fluor 488 (A32723; RRID: AB_2633275), goat anti-rabbit secondary antibody Alexa Fluor 488 (A32731; RRID: AB_2633280), goat anti-mouse secondary antibody Alexa Fluor 555 (A32727; RRID: AB_2633276), goat anti-rabbit secondary antibody Alexa Fluor 555 (A21435; RRID: AB_2535856) and goat anti-guinea pig secondary antibody Alexa Fluor 633 (A21105; RRID:AB_2535757). After the samples were rinsed thrice with PBS and then washed thrice for 15 min with PBT, they were mounted on glass slides using FluorSave reagent (Merck Millipore, #345789). Quantification of immunostaining signals was conducted using the ImageJ software version 1.53q (Schneider et al., 2012).

### Connectome analysis

The connectome analysis was performed using NeuronBridge (Clements et al., 2020; Jenett et al., 2012; Meissner et al., 2023; Otsuna et al., 2018 preprint) (https://neuronbridge.janelia.org/) and neuPrint+ (Plaza et al., 2022; Scheffer et al., 2020) (https://neuprint.janelia.org/). NeuronBridge is a database in which neurons labeled by *GAL4* drivers are mapped after identification using light and electron microscopic connectome analysis. The NeuronBridge body IDs corresponding to two of the three pairs of CA-LP neurons (Fig. S2A) were #295063181 and #5813067334. Neurons connected with #295063181 and #5813067334 were identified in the connectome database on neuPrint+, and the names of the connected neurons and data of the connection numbers were obtained. The percentages of neurons indicate the proportions of each clock neuron in relation to the whole connection to #295063181 and #5813067334 (Fig. S2A).

### Counting mature egg numbers in ovaries

The ovaries of virgin females were dissected in PBS. The numbers of mature eggs (stage-14 oocytes) (King, 1970) in the ovaries were counted under a stereomicroscope (Leica MZ10F).

### Forced activation of CA-LP neurons by *TrpA1* overexpression

Flies carrying *R94H10-GAL4* and two copies of *UAS-TrpA1* were reared at 21°C from embryos to newly eclosed adults. Upon eclosion, flies were randomly assigned to two groups: one maintained at 21°C (permissive temperature) where *TrpA1* was not activated, and the other raised at 29°C (restrictive temperature) to activate *TrpA1*. After 4 days, mature egg numbers were counted.

### Generation of a *UAS-Pink Flamindo* transgenic line

The pcDNA3.1 plasmid containing the Pink Flamindo coding sequence (Pink Flamindo-pcDNA3.1; Harada et al., 2017) was obtained from Addgene (plasmid #102356). The following primers were used for PCR amplification, to obtain the Pink Flamindo coding sequences with *EcoR*I and *Xba*I sites at the 5′ and 3′ termini, respectively: Pink Flamindo F (5′-ACTGAATTCATGCTGGTGAGCAAGGGC-3′) and Pink Flamindo R (5′-CCTGCTCGACATGTTCATTAGATCTCAG-3′). PCR products were digested with *EcoR*I and *Xba*I, purified, and ligated with the *EcoR*I-*Xba*I-digested pWALIUM10-moe plasmid (Perkins et al., 2015). Transformants were generated using the phiC31 integrase system in the *P{CaryP}attP2* strain (Groth et al., 2004) by WellGenetics. *w*+ transformants of pWALIUM10-moe were established using standard protocols.

### cAMP imaging in the CA after the administration of DH31 peptide

In the implementation of live imaging in the CA, experimental conditions were optimized with reference to previous studies (Lee et al., 2017; Meiselman et al., 2017). cAMP transients in the CA were imaged in flies expressing *UAS-Pink Flamindo* and *UAS-mCD8::GFP* driven by *JHAMT-GAL4*. For preparing non-dormant flies, newly eclosed virgin females (0-6 h after eclosion) were cultured at 25°C under a 12 h light/12 h dark cycle for 1 day. For preparing dormant flies, newly eclosed virgin females were cultured at 11°C under a SD cycle for 12 days. Adult brain-CA-gut complexes were dissected in Schneider's *Drosophila* Medium (SDM; Thermo Fisher Scientific, #21720024) without supplementation with fetal bovine serum. The brain-CA-gut complexes were then treated with 100 μl of collagenase solution (0.05 mg/ml collagenase in SDM; Sigma-Aldrich, #C0130) with gentle rotation for 9 min at 25-27°C and vortexed gently for 1 min, ensuring that the brain and gut were not physically separated. The samples were then washed twice with 1 ml of SDM and once with 500 μl of SDM. The dissected tissues were held in a glass-bottom dish (35×10 mm, IWAKI, #3910-035) with an insect pin (ϕ0.10 mm, Ento Sphinx Insect Pins) and silicone grease (Beckman), and 20 μl of SDM was added to cover the tissue. Live imaging was performed at 25-27°C using a laser scanning confocal microscope (LSM700, Carl Zeiss) with a 20× objective lens. mCD8::GFP and Pink Flamindo were excited with 488 nm and 555 nm lasers, respectively. Time-lapse images were acquired every 8 s for 472 s. Then, 112 s after starting live-imaging, 80 μl of SDM with or without 375 nM synthetic DH31 peptide (NH_2_-TVDFGLARGYSGTQEAKHRMGLAAANFAGGP-CONH_2_, synthesized by Eurofins Genomics; final concentration: 300 nM) or 125 μM NKH477 (Sigma-Aldrich, #N3290; final concentration: 100 μM) was applied on the tissue. For image processing, the CA was selected in a region of interest (ROI) over multiple time frames. The mean fluorescence intensities were measured along the time axis using the ImageJ software version 1.53q (Schneider et al., 2012). Data were analyzed using Microsoft Excel.

### cAMP imaging in the CA with bath-applied ATP-dependent neuronal activation through P2X2

Newly eclosed virgin females, which carry transgenes of *JHMAT-GAL4*, *UAS-Pink Flamindo*, *R18G01-LexA* and *LexAop-P2X2*, were cultured at 11°C under a SD cycle for 12 days. All experimental procedures were almost the same as those used for cAMP imaging in the CA with the administration of the DH31 peptide; except for the following two points. First, the brain-CA-gut complexes were not treated with collagenase. Second, the DH31 peptide was not used: 112 s after starting live-imaging, 80 μl of SDM with or without 3.125 mM ATP (Promega #E6011; final concentration: 2.5 mM) was applied on the tissue. After live imaging, the vehicle- and ATP-treated brain-CA-gut complexes were collected and used for immunostaining experiments with anti-DH31 antibody. Of note, the immunoreactive signals from anti-DH31 antibody were diminished in DH31 loss-of-function mutant animals (Fig. 3A).

### Measurement of JH III titers

Newly emerged virgin females were collected within 6 h of eclosion and reared on standard medium at 11±0.5°C under SD conditions for 6 days. Forty adult female flies of each genotype were punctured using a tungsten needle and placed in a plastic tube with a hole at the bottom. The tube was then connected to a silanized glass vial (GL Science, #5183-4507) and centrifuged at 9100 ***g*** for 5 min. Pre-cooled PBS (150 µl) was added to the glass vial where the hemolymph was collected, and 6.25 pg/µl (in acetonitrile) of JH III-D3 (Toronto Research Chemicals, #E589402) was added as an internal control. Subsequently, 600 µl of hexane was added, and the samples were stirred for 1 min. The samples were then centrifuged at 2000 ***g*** for 5 min at 4°C, and 500 µl of the organic phase was transferred to a fresh silanized vial. The samples were dried under a gentle nitrogen flow and stored at −20°C until further analysis. JH titers from these hemolymph extracts were determined using LC-MS/MS as previously described (Ramirez et al., 2020).

### Reverse transcription-quantitative PCR (RT-qPCR)

Total RNA was extracted from whole bodies of 6-day-old adult virgin female flies (under a SD cycle) after ∼4-6 h of light period. RNA was reverse-transcribed using ReverTra Ace qPCR RT Master Mix with gDNA Remover (Toyobo). Synthesized cDNA samples were used as templates for quantitative PCR using THUNDERBIRD SYBR qPCR Mix (Toyobo) on a Thermal Cycler Dice Real Time System (Takara Bio). The amount of target RNA was normalized to the endogenous control *ribosomal protein 49* gene (*rp49*) and the relative fold change was calculated. The expression levels of *yolk protein 1*, *yolk protein 2* and *yolk protein 3* were compared using the ΔΔCt method. The following primers were used for this analysis: rp49 F (5′-CGGATCGATATGCTAAGCTGT-3′), rp49 R (5′-GCGCTTGTTCGATCCGTA-3′), Dh31-R F (5′-TACATCCTTACGCCCTTTCGTCCT-3′), Dh31-R R (5′-GGCAACGCACAGACCTTGAAATGA-3′) (Goda et al., 2018), yolk protein1 F (5′-CAGGCTCAGTACACCCACAC-3′), yolk protein1 R (5′-CTCAACGTTGTGGTGGATCTG-3′), yolk protein2 F (5′-ACCCTTAAGAAGCTGCAGGAG-3′), yolk protein2 R (5′-ATGGTTGAACTGGGACAGATG-3′), yolk protein3 F (5′-CTCAAGAGCAGCGACTACGAC-3′) and yolk protein3 R (5′-TAGCGTTTGAAGTTGGTCAGG-3′).

### Statistical analysis

All experiments were performed independently at least twice. The sample sizes were chosen based on the number of independent experiments required for statistical significance and technical feasibility. The experiments were not randomized, and the investigators were aware of which samples had received which treatment. All raw quantitative data are provided in Tables S1 and S2. All statistical analyses were performed using the ‘R’ software version 4.0.3. Details of the statistical analyses are described in figure legends.

## Supplementary Material

10.1242/develop.201186_sup1 Supplementary information
