# Supplementary material for: Female reproductive dormancy in Drosophila is regulated by DH31-producing neurons projecting into the corpus allatum
Source: Development. 2023 May 23;150(10):dev201186. doi: 10.1242/dev.201186 (PMC10233717; doi:10.1242/dev.201186)
Supplement: Supplementary information [file develop-150-201186-s1.pdf]

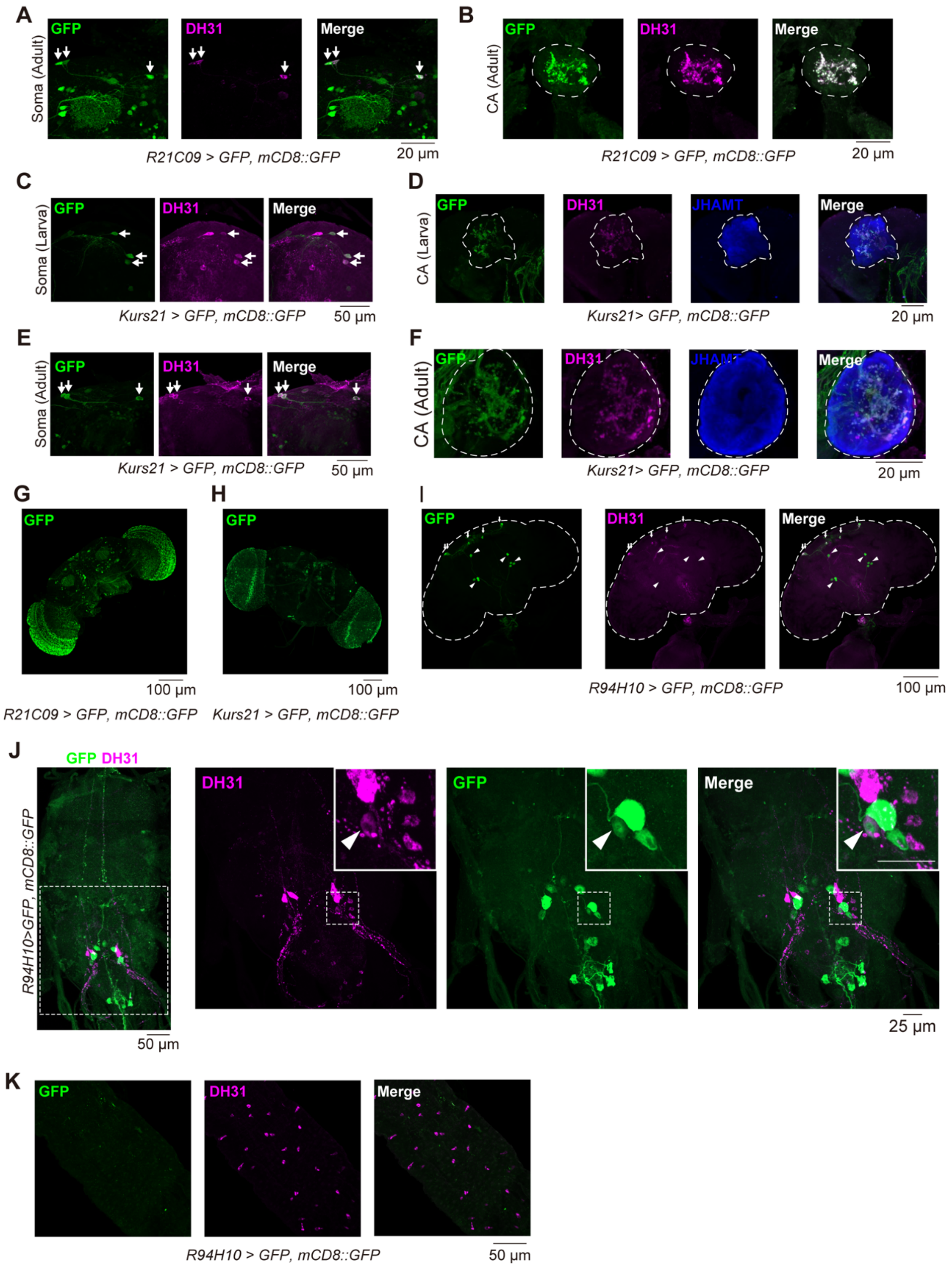

Fig. S1. *R21C09-GAL4* and *Kurs21-GAL4* are active in DH31$_{CA}$ neurons. (A, B) GFP signals in the soma (A) and CA region (B) of an adult female *R21C09-GAL4 UAS-GFP UAS-mCD8::GFP*. Samples were immunostained with

Development • Supplementary information

anti-GFP (green) and anti-DH31 (magenta) antibodies. Arrows indicate the soma of $DH31_{CA}$ neurons and dashed lines outline the CA.

(C, D) GFP signals in the soma (C) and CA region (D) of a larva of *Kurs21-GAL4 UAS-GFP UAS-mCD8::GFP*. (C) Immunostaining with anti-GFP (green) and anti-DH31 (magenta) antibodies. Arrows indicate the soma of $DH31_{CA}$ neurons. (D) Immunostaining with anti-GFP (green), anti-DH31 (magenta), and anti-JHAMT (blue) antibodies.

(E, F) GFP signals in the soma (E) and CA regions (F) of an adult female *Kurs21-GAL4 UAS-GFP UAS-mCD8::GFP*. (E) Immunostaining with anti-GFP (green) and anti-DH31 (magenta) antibodies. Arrows indicate the soma of $DH31_{CA}$ neurons. (F) Immunostaining with anti-GFP (green), anti-DH31 (magenta), and anti-JHAMT (blue) antibodies.

(G, H) Lower magnification views of *R21C09-GAL4 UAS-GFP UAS-mCD8::GFP* (G) and *Kurs21-GAL4 UAS-GFP UAS-mCD8::GFP* (H). Samples were immunostained with an anti-GFP antibody (green).

(I) Transgenic visualization of $DH31_{CA}$ neurons by GFP driven by *R94H10-GAL4*. The sample is the same as that shown in Fig. 1D, while signals were visualized with anti-GFP (green) and anti-DH31 (magenta) antibodies. Note that some *R94H10-GAL4*-positive neurons marked with arrowheads, which do not innervate the CA, were DH31-negative.

(J) Immunostaining signal with anti-GFP (green) and anti-DH31 (magenta) antibodies in the ventral nerve cord of an adult female of *R94H10-GAL4 UAS-GFP UAS-mCD8::GFP*. White arrowheads indicate the *R94H10-GAL4*- and DH31-double positive VNC neurons.

(K) Immunostaining signal with anti-GFP (green) and anti-DH31 (magenta) antibodies in the posterior midgut of an adult female of *R94H10-GAL4 UAS-GFP UAS-mCD8::GFP*.

Development • Supplementary information

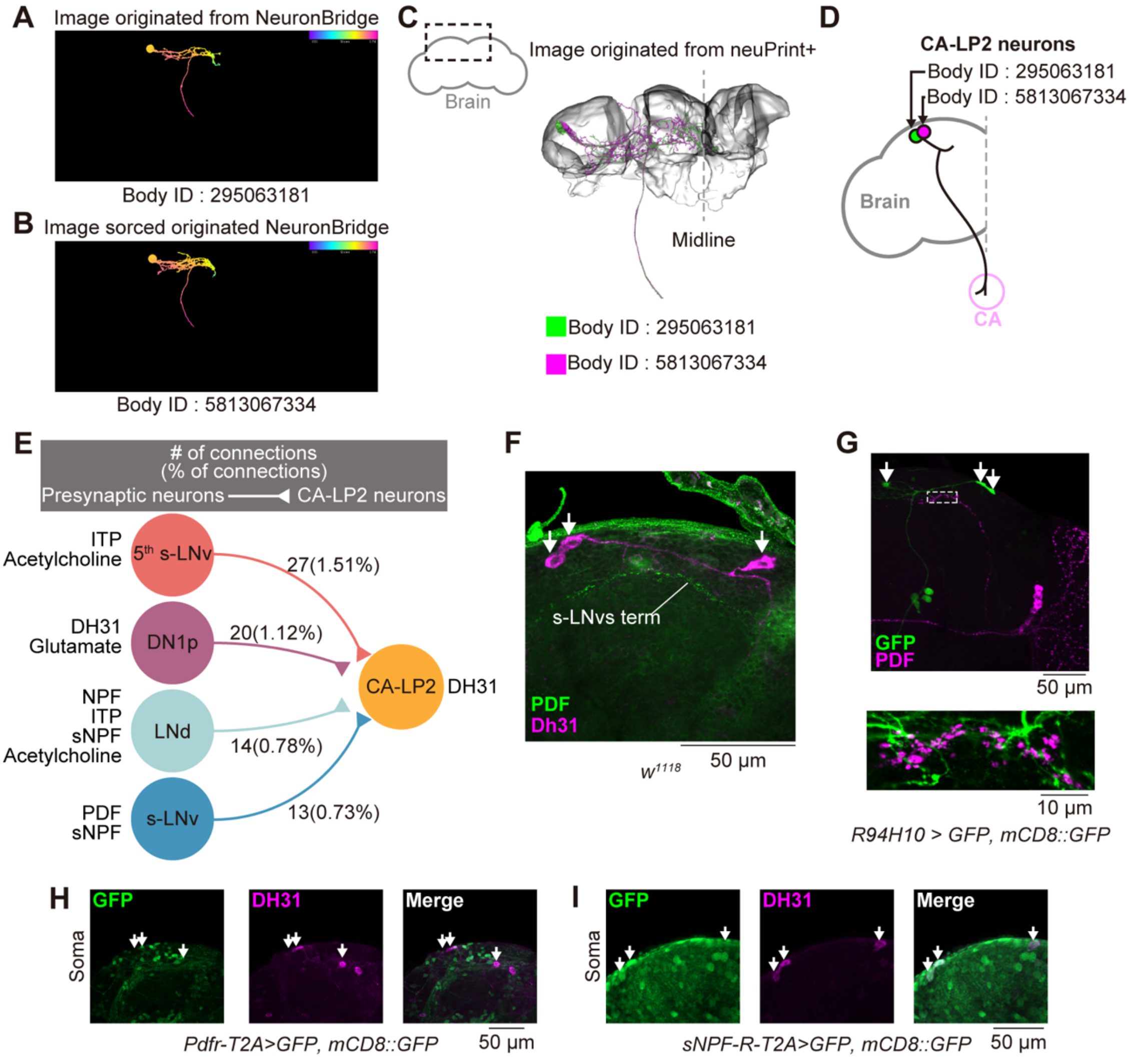

Fig. S2. A subset of circadian clock neurons innervates the CA-LP neurons

In all panels, arrows indicate the cell bodies of CA-LP neurons.

(A, B) Computationally-reconstructed images of the two pairs of $DH31_{CA}$ neurons. These images were generated using NeuronBridge (96–98) and deposited in the publically available website (https://neuronbridge.janelia.org/). We used the reconstructed image of Body IDs 29506318 (A) and 5813067334 (B) under the condition of Creative Commons License CC-BY 4.0. Relative positions of cell bodies and neurites are represented in color bar scales, in which a surface or a deep area of the brain are represented with blue and red, respectively.

(C) A computationally-merged reconstructed image of Body IDs 29506318 (green) and 5813067334 (magenta). The image on the right side, generated

Development • Supplementary information

using neuPrint+ (99, 100), was derived from the publically available website (https://neuprint.janelia.org/?dataset=hemibrain:v1.2&qt=findneurons). This neuPrint+ image corresponds to the dorsolateral and dorsomedial brain region, marked by dashed lines in the whole brain illustration (left side). We used the image under the condition of Creative Commons License CC-BY 4.0.

(D) A schematic representation of the relative positions of Body IDs 29506318 (green) and 5813067334 (magenta) in the brain hemisphere based on C. Considering the positions of the cell bodies; we concluded that the neurons of Body IDs 29506318 and 5813067334 correspond to the CA-LP2 neurons.

(E) Connectivity diagram between CA-LP and clock neurons (s-LNv, 5th s-LNv, DN1p, and LNd). Numbers outside parentheses indicate contact site counts marked by presynaptic densities. Numbers in parentheses indicate the percentage of contact site counts with each clock neuron population per total contact between CA-LP neurons and all the neurons described in neuPrint+. Neuropeptides and neurotransmitters produced in the clock neurons are also indicated.

(F) Immunostaining with anti-PDF (green) and anti-DH31 (magenta) antibodies in the brain region, including the soma of DH31$_{CA}$ neurons (arrows), of wild-type (*w^{1118}*) adult females. Axonal termini of s-LNv neurons (s-LNv term) are indicated by ‘s-LNvs term.’

(G) Immunostaining with anti-GFP (green) and anti-PDF (magenta) antibodies in an adult female expressing GFP driven by *R94H10-GAL4*. The lower panel represents a high magnification, with increased gains in the dashed inset in the upper panel. Note that the axonal termini of PDF clock neurons are in close proximity to the neuronal processes of CA-LP neurons.

(H, I) Expression of *Pdfr* (H) and *sNPF-R* (I) visualized using *Pdfr-T2A-GAL4*- and *sNFPR-T2A-GAL4*-driven GFP, respectively (green). The soma of the CA-LP neurons is marked with an anti-DH31 antibody (magenta).

Development • Supplementary information

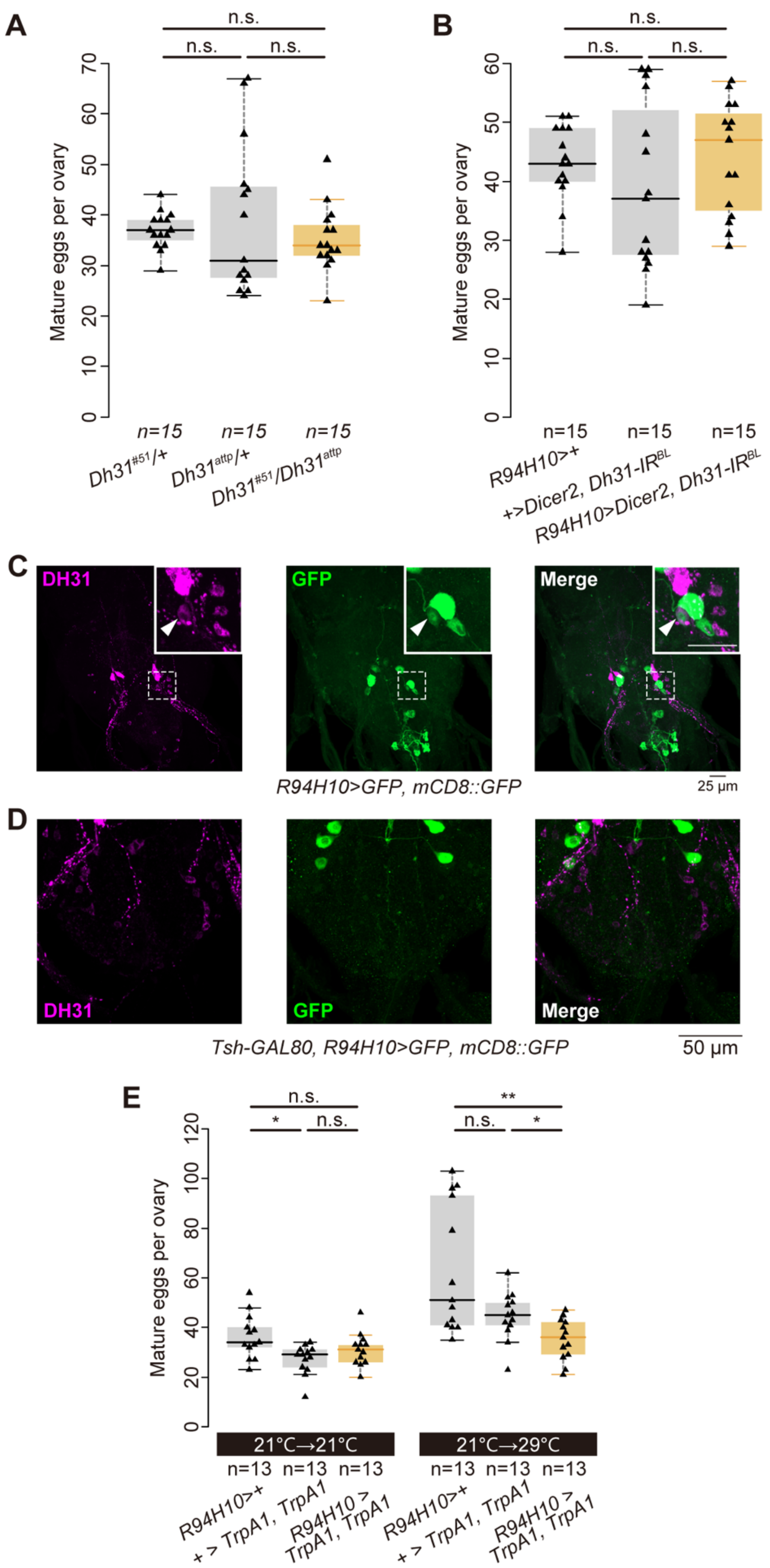
A
n.s.
n.s.
n.s.
Mature eggs per ovary
n=15
n=15
n=15
Dh31#51/+
Dh31attp/+
Dh31#51/Dh31attp
B
n.s.
n.s.
n.s.
Mature eggs per ovary
n=15
n=15
n=15
R94H10>+
+>Dicer2, Dh31-IRBL
R94H10>Dicer2, Dh31-IRBL
C
DH31
GFP
Merge
R94H10>GFP, mCD8::GFP
25 µm
D
DH31
GFP
Merge
Tsh-GAL80, R94H10>GFP, mCD8::GFP
50 µm
E
n.s.
*
n.s.
**
n.s.
*
Mature eggs per ovary
21°C→21°C
21°C→29°C
n=13 n=13 n=13
n=13 n=13 n=13
R94H10>+
+ > TrpA1, TrpA1
R94H10 > TrpA1, TrpA1
R94H10>+
+ > TrpA1, TrpA1
R94H10 > TrpA1, TrpA1

Fig. S3. Some supplemental analyses on *Dh31* and CA-LP neurons

Samples were derived from virgin females 4 d after eclosion under non-dormancy-inducing conditions.

(A, B) Quantification of mature eggs in *Dh31* genetic mutant females (A) and *R94H10-GAL4*-driven *Dh31* RNAi females (B) under non-dormancy-inducing conditions.

(C, D) Immunostaining signal with anti-GFP (green) and anti-DH31 (magenta) antibodies in the caudal part of the ventral nerve cord of an adult female of *R94H10-GAL4 UAS-GFP UAS-mCD8::GFP* (C) and *R94H10-GAL4 tsh-GAL80 UAS-GFP UAS-mCD8::GFP* (D). Note that the images in C are the exact copy of a part of Fig. S1J to make it easier to compare the images in D. White arrowheads indicate the *R94H10-GAL4*- and DH31-double positive VNC neurons.

(E) Quantification of mature eggs in *R94H10-GAL4*-driven *TrpA1* overexpression females that were shifted to permissive temperature (left) and to restrictive temperature (right)

Statistical analysis: Wilcoxon rank sum test with Bonferroni’s correction.

**P*<0.05 and ***P* <0.01. n.s.: not significant.

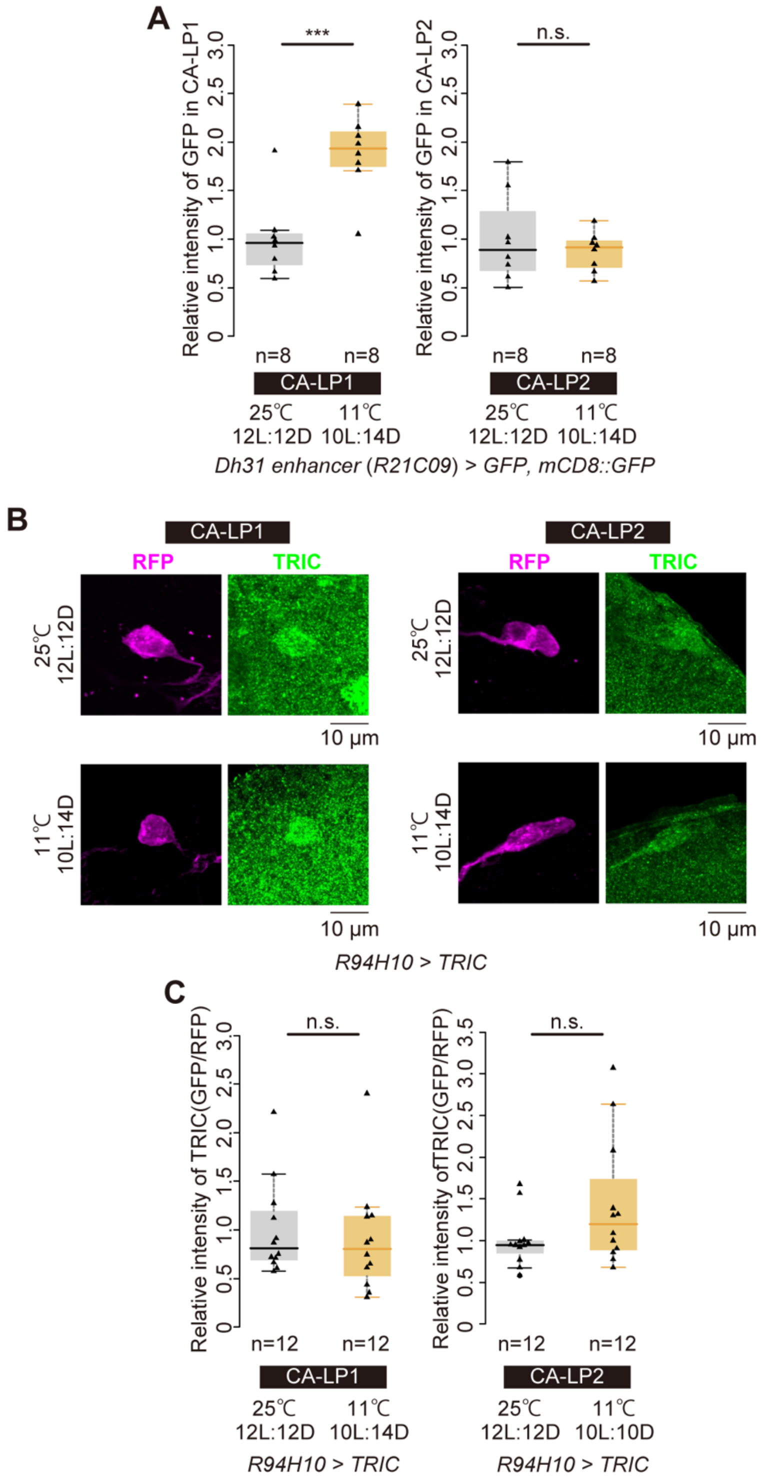

Fig. S4. DH31 protein levels, *Dh31* gene expression levels, and TRIC signals in CA-LP neurons

Samples were derived from virgin females 12 d after eclosion under dormancy- and non-dormancy-inducing conditions. For all analyses, non-dormancy-

Development • Supplementary information

inducing conditions were 25 ºC with 12:12 light/dark, and dormancy-inducing conditions were 11 ºC with 10:14 light/dark.

(A) Quantitative comparison of anti-GFP immunoreactivity in the soma of CA-LP1 and CA-LP2 neurons with *Dh31 enhancer* (*R21C09*)-*GAL4*-driven mCD8::GFP between non-dormancy- and dormancy-inducing conditions.

(B, C) TRIC signals in CA-LP1 and CA-LP2 neurons. The TRIC construct was driven by *R94H10-GAL4*. (B) Representative images of anti-RFP (magenta) and anti-GFP immunoreactivity (TRIC; green) signals in non-dormancy- and dormancy-inducing conditions. (C) Quantitative comparison of TRIC signals normalized by RFP signals in CA-LP1 and CA-LP2 neurons between non-dormancy- and dormancy-inducing conditions.

Statistical analysis: Student's *t*-test. *$P<0.05$ and ***$P<0.001$. n.s.: not significant.

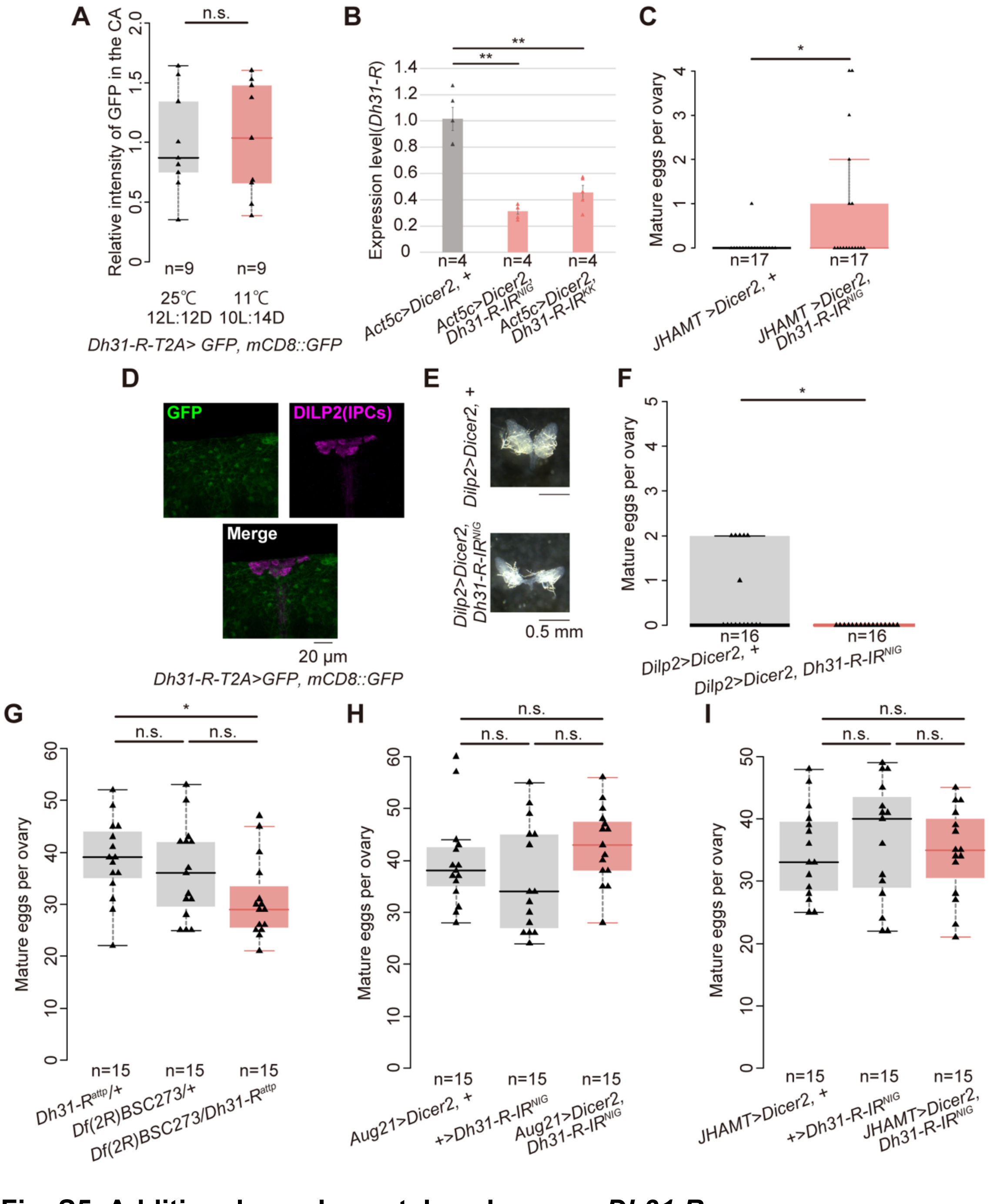

Fig. S5. Additional supplemental analyses on *Dh31-R*

Samples were derived from virgin females 12 d after eclosion under dormancy- (A–F) and non-dormancy- (A) inducing conditions, and 4 d after eclosion under dormancy-inducing conditions (G–I).

(A) Quantitative comparison of anti-GFP immunoreactivity in the soma of CA-LP1 and CA-LP2 neurons of *Dh31-R-T2A-GAL4*-driven mCD8::GFP between non-dormancy- and dormancy-inducing conditions.

Development • Supplementary information

(B) Quantification of *Dh31-R* mRNA in control and *Actin5C-GAL4*-driven *Dh31-R* RNAi virgin females under dormancy-inducing conditions by RT-qPCR. Two independent RNAi lines, *Dh31-R-IR*^{KK} and *Dh31-R-IR*^{NIG} were used. Values are presented as means ± SE.

(C) Quantification of mature eggs per ovary in control and *JHAMT-GAL4*-driven *Dh31-R* RNAi virgin females under dormancy-inducing conditions.

(D) Immunostaining of anti-GFP (green) and anti-Dilp2 (magenta) antibodies in the insulin-producing cells (IPCs) of *Dh31-R-T2A-GAL4*-driven *mCD8::GFP* virgin females under dormancy-inducing conditions.

(E, F) Mature egg formation in control and *Dilp2-GAL4*-driven *Dh31-R* RNAi virgin females under dormancy-inducing conditions. (E) Representative images of the ovaries. (F) Quantification of mature eggs per ovary.

(G–I) Quantification of mature eggs in *Dh31-R* genetic mutant females (G), *Aug21-GAL4*-driven (H), and *JHAMT-GAL4*-driven (I) *Dh31-R* RNAi females under non-dormancy-inducing conditions.

Statistical analysis: Student's *t*-test for A, Tukey–Kramer's HSD test for B, and Wilcoxon rank sum test for C, F, G, H, and I with Bonferroni's correction. *P<0.05 and **P <0.01. n.s.: not significant.

Development • Supplementary information

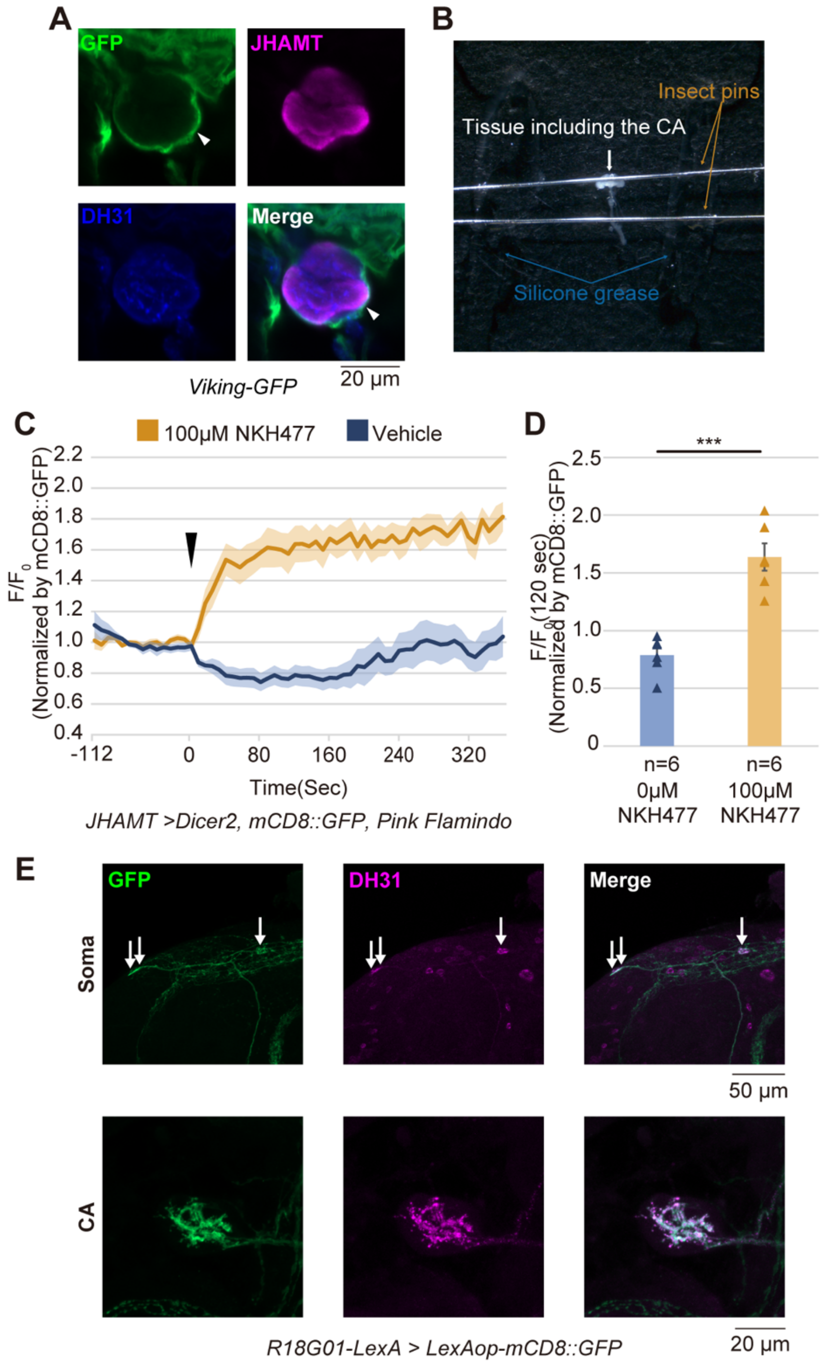

Fig. S6. Pink Flamindo imaging in the CA.

Supplementary data for Fig. 5.

(A) A representative photograph demonstrating how to mount the dissected, unfixed tissue containing the CA. To keep the tissue immobile, two insect pins were placed on the tissue, being careful not to hide the CA. To fix the position of the insect pins, silicon grease is applied to the bottom of the dish.

Development • Supplementary information

(B) GFP signal (green) in the CA of *Viking-GFP* strain that produces a functional GFP-tagged version of the Viking protein. The sample was immunostained with anti-JHAMT (magenta) and anti-DH31 (blue) antibodies. The arrowhead indicates the presence of a collagen layer surrounding the CA.
(C, D) Changes in the relative fluorescence intensity of *JHAMT-GAL4*-driven Pink Flamindo in the CA. (C) Pink Flamindo signals with or without stimulation by 100 µM NKH477 (each n = 6). F/F_0 values are normalized by signal intensity of *JHAMT-GAL4*-driven mCD8::GFP. Samples were derived from virgin females 0–24 h after eclosion under non-dormancy-inducing conditions. (D) Quantification of normalized Pink Flamindo signals in the CA at 120 s after the stimulation.
(E) Immunostaining signal with anti-GFP (green) and anti-DH31 (magenta) antibodies in the brain region, including the soma of CA-LP neurons (arrows), and in the CA region in an adult female of *R18G01-LexA LexAop-mCD8::GFP*.
Values in C and D are presented as mean ± SE. Statistical analysis: Student's *t*-test for D. ****P* <0.001. n.s.: not significant.

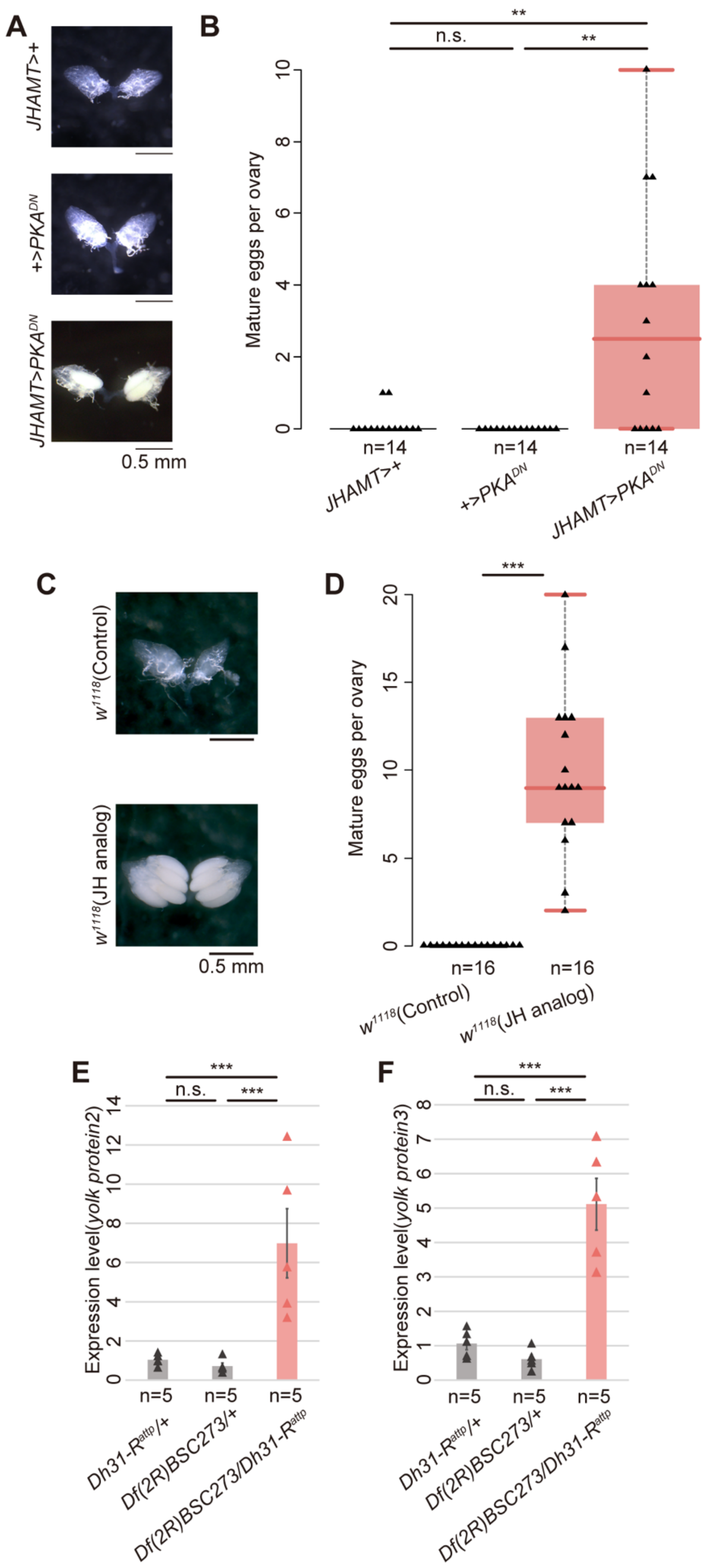
A
JHAMT>+
+>PKA^DN
JHAMT>PKA^DN
0.5 mm
B
**
n.s.
**
Mature eggs per ovary
n=14
n=14
n=14
JHAMT>+
+>PKA^DN
JHAMT>PKA^DN
C
w1118(Control)
w1118(JH analog)
0.5 mm
D
***
Mature eggs per ovary
n=16
n=16
w1118(Control)
w1118(JH analog)
E
***
n.s.
***
Expression level(yolk protein2)
n=5
n=5
n=5
Dh31-Rattp/+
Df(2R)BSC273/+
Df(2R)BSC273/Dh31-Rattp
F
***
n.s.
***
Expression level(yolk protein3)
n=5
n=5
n=5
Dh31-Rattp/+
Df(2R)BSC273/+
Df(2R)BSC273/Dh31-Rattp

Fig. S7. Mature egg formation in dominant negative PKA overexpressors and JH-treated animals; and mRNA levels of *yolk protein 2* and *yolk protein 3* in *Dh31-R* genetic mutants under dormancy-inducing conditions. Samples were derived from virgin females 6 d (for E and F) or 12 d (for A–D) after transfer to dormancy-inducing conditions.

(A, B) Mature egg formation in control and CA-specific *dominant negative PKA* (*PKA^{DN}*)-overexpressing females under dormancy-inducing conditions. (A) Representative images of the ovaries. (B) Quantification of mature eggs per ovary.

(C, D) Mature egg formation in wild-type (*w^{1118}*) adult females, with or without oral administration of methoprene (JH analog, JHA) under dormancy-inducing conditions. (C) Representative images of the ovaries. (D) Quantification of mature eggs per ovary.

(E, F) Quantification of mRNA of *yolk protein 2* (E) and *yolk protein 3* (F) in control and *Dh31-R* genetic mutant females under dormancy-inducing conditions by RT-qPCR. Values in E and F are presented as means ± SE. Statistical analysis: Wilcoxon rank sum test for B and D; Tukey–Kramer's HSD test for E and F. **P<0.01***P <0.001. n.s.: not significant.

Development • Supplementary information

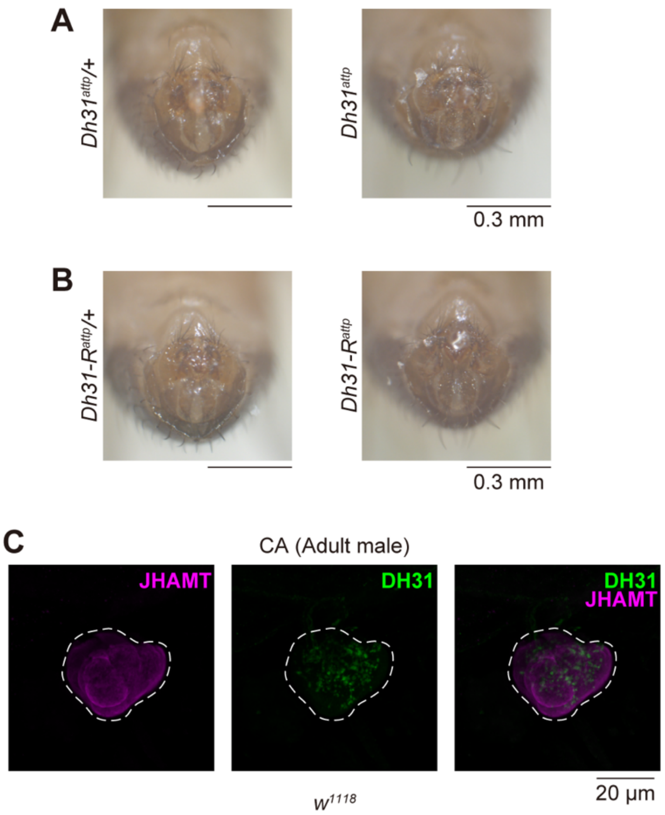

Fig. S8. Male genitalia of loss of *Dh31* and *Dh31-R* genetic mutants, and $DH31_{CA}$ neurons in the adult male.

(A, B) Male genitalia of loss of *Dh31* (A, right) and *Dh31-R* (B, right) genetic mutants. Control heterozygous animals are shown in the left side.

(C) Immunostaining signal with anti-DH31 antibody (green) along with anti-JHAMT antibody to visualize the CA (magenta) of wild-type (*w^{1118}*) adult male. Note that DH31-positve puncta are observed in the CA region.

Development • Supplementary information

Table S1. Raw datasets for main figures

The separate file includes mature egg numbers for Figs. 3 and 4, anti-DH31 signal intensities for Fig. 3 and 5, PinkFlamindo signal intensities for Fig. 5, hemolymph JH III titers for Fig. 6, and RT-qPCR data for Fig. 6.

Click here to download Table S1

Table S2. Raw datasets for supplemental figures.

The separate file includes neuPrint+ body IDs of the neurons connecting CA-LP2 neurons for Fig. S2, mature egg numbers for Figs. S3, S5 and S7, *Dh31 enhancer-GAL4* driven GFP signal intensities for Fig. S4, TRIC signal intensities for Fig. S4, RT-qPCR data for Fig. S5 and S7, *Dh31-R-T2A-GAL4* driven GFP signal intensities for Fig. S5, RT-qPCR data for Fig. S5 and S8, and PinkFlamindo signal intensities for Fig. S6.

Click here to download Table S2
