# Supplementary material for: Female reproductive dormancy in Drosophila is regulated by DH31-producing neurons projecting into the corpus allatum
Source: Development. 2023 May 23;150(10):dev201186. doi: 10.1242/dev.201186 (PMC10233717; doi:10.1242/dev.201186)
Supplement: Supplementary file 1 [file develop-150-201186_review_history.pdf]

Female reproductive dormancy in *Drosophila* is regulated by DH31-producing neurons projecting into the *corpus allatum*

Yoshitomo Kurogi, Eisuke Imura, Yosuke Mizuno, Ryo Hoshino, Marcela Nouzova, Shigeru Matsuyama, Akira Mizoguchi, Shu Kondo, Hiromu Tanimoto, Fernando G. Noriega and Ryusuke Niwa
DOI: 10.1242/dev.201186

Editor: Irene Miguel-Aliaga

Review timeline

Original submission:	3 August 2022
Editorial decision:	8 September 2022
First revision received:	4 March 2023
Editorial decision:	28 March 2023
Second revision received:	3 April 2023
Accepted:	12 April 2023

Original submission

First decision letter

MS ID#: DEVELOP/2022/201186

MS TITLE: Female reproductive dormancy in *Drosophila* is regulated by DH31-producing neurons projecting into the *corpus allatum*

AUTHORS: Yoshitomo Kurogi, Eisuke Imura, Yosuke Mizuno, Ryo Hoshino, Marcela Nouzova, Shigeru Matsuyama, Akira Mizoguchi, Shu Kondo, Hiromu Tanimoto, Fernando G Noriega, and Ryusuke Niwa

I have now received all the referees' reports on the above manuscript, and have reached a decision. The referees' comments are appended below, or you can access them online: please go to BenchPress and click on the 'Manuscripts with Decisions' queue in the Author Area.

As you will see, the referees express considerable interest in your work, but have some significant criticisms and recommend a substantial revision of your manuscript before we can consider publication. If you are able to revise the manuscript along the lines suggested, which may involve further experiments, I will be happy receive a revised version of the manuscript. Your revised paper will be re-reviewed by one or more of the original referees, and acceptance of your manuscript will depend on your addressing satisfactorily the reviewers' major concerns. Please also note that Development will normally permit only one round of major revision. If it would be helpful, you are welcome to contact us to discuss your revision in greater detail. Please send us a point-by-point response indicating your plans for addressing the referee's comments, and we will look over this and provide further guidance.

Please attend to all of the reviewers' comments and ensure that you clearly highlight all changes made in the revised manuscript. Please avoid using 'Tracked changes' in Word files as these are lost in PDF conversion. I should be grateful if you would also provide a point-by-point response detailing how you have dealt with the points raised by the reviewers in the 'Response to Reviewers' box. If you do not agree with any of their criticisms or suggestions please explain clearly why this is so.

© 2023. Published by The Company of Biologists under the terms of the Creative Commons Attribution License (https://creativecommons.org/licenses/by/4.0/).

Reviewer 1

Advance summary and potential significance to field

The study by Kurogi et al addressed an important question of how unfavorable environmental conditions regulate female reproduction. It has been well established that Drosophila females will enter a state of reproductive dormancy in the winter when environmental temperature is lower and the daily period of light is shortened. Furthermore, reproductive dormancy in insects is associated with a lower titer of juvenile hormone (JH) released from the corpora allata (CA). Here, the authors claimed that they have found the elusive brain-to-CA connection that regulates female reproductive dormancy through controlling JH.
They first identified a population of Dh31-expressing brain neurons called CA-LP2 that send axons to the CA and found that Dh31 receptor is expressed in the CA.
Second, the authors showed that Dh31 expression in the CA-LP2 neurons and Dh31R expression in the CA are both required for the shrinking of the ovaries and the lower titer of juvenile hormone under the extreme environmental condition.
Finally, using an imaging system to measure intracellular cAMP concentration they showed that CA cells exhibited an increase of cAMP in response to Dh31, and this response requires the expression of D31R in CA cells. Overall, these are all interesting results supporting a role of CA-LP2 neurons in regulating the CA.
However, crucial evidence is still missing to support the claim that the CA-LP2 neurons play an essential role in regulating temperature-dependent reproductive dormancy. In other words, the context for Dh31 signaling to regulate female reproduction is unclear and the authors have yet to demonstrate environmental temperature is this context.

Comments for the author

Major concerns:
1. Although perturbing Dh31 signaling is able to change JH titer and ovary morphology under the extreme environmental condition, it is a different thing to say that the communication between the brain and the CA via Dh31 is the major pathway for environmental condition to change the reproductive status. The authors have not shown what has been changed by the extreme environmental condition. Does environmental condition change the level of Dh31, Dh31R or the excitability of the CA-LP2 neurons? Looking at the functional role of the CA-LP2 neurons, Dh31 and Dh31R in females under normal environmental conditions (25 degree and 12:12 light condition) may provide an opportunity to compare and contrast in order to identify the factor(s) that change with the environmental conditions. For example, a recent published study (Meiselman et al, 2022) showed that AstC expression is temperature dependent. However, the AstC-expressing clock neurons DN3 do not seem to synapse with the CA-LP neurons (Figure 2). As such, it is possible that DN3 is part of a different pathway to regulate reproductive dormancy. It is therefore imperative for the authors to resolve these outstanding issues.

2. Related to the above concern, the authors need to be forthcoming with the conditions for the imaging experiments presented in Figure 5. Lines 591-592 in Methods indicate that for these imaging experiments, CA were obtained from newly eclosed virgin females. These are not females under low-temperature and short light exposure. Therefore, it is possible that Dh31 would not trigger cAMP increases in females under reproductive dormancy. Furthermore, collagenase treatment can also dissolve synaptic connections. A more physiologically relevant experiment is to stimulate the CA-LP2 neurons, perhaps with optogenetics or the ATP-P2X2 system, while imaging the CA. With this experimental regimen, one can determine whether Dh31 signaling is required for the change of intracellular cAMP and whether CA-LP2 is the major conduit linking environmental conditions to CA function.

3. It is counterintuitive to think that an increase of intracellular cAMP concentration would reduce juvenile hormone release. The authors would need to demonstrate this empirically, perhaps by perturbing adenylyl cyclase or phosphodiesterase in the AC.

4. Figure 3. According to Janelia Flylight, R94H10 is also expressed in VNC not just CA-LP. The authors should perform experiments to assess whether VNC neurons are relevant.

© 2023. Published by The Company of Biologists under the terms of the Creative Commons Attribution License (https://creativecommons.org/licenses/by/4.0/).

Minor concerns:
1. The upstream regulators of CA-LP are not well characterized. Due to the insufficient evidence to support the notion that the clock neurons are functionally upstream of CA-LP, I would suggest this figure to be a supplementary figure. Specifically, the indicated neurons as well as the neuropeptides or neurotransmitters in Fig 2A are not experimentally validated to be upstream of CA-LP2. A counter example is provided by the authors that the PDF receptor is not expressed in CA-LP. Fig 2A gives the impression that all indicated neuropeptides and neurotransmitters are utilized to regulate CA-LP2. This may be misleading the readers. Furthermore, it is unclear whether sNPF receptor is relevant to the dormancy phenotype. To support this, it will be necessary to perform genetic perturbation experiments, such as knocking down sNPF receptor in CA-LP, to demonstrate that clock neurons are upstream regulators of CA-LP.

Reviewer 2

Advance summary and potential significance to field

This paper is the first to show that a defined set of neurons control the production of JH in response to environmental conditions and thus mediate the adult diapause of Drosophila. This paper provides a firm basis for further elucidation of how environmental signals can disrupt the normal endocrine control of egg maturation. It also should stimulate the search in other species for a similar role for the same peptide or other peptides with the same function.

Comments for the author

Although it has long been known that neurosecretory neurons in the pars lateralis (PL) control the secretion of juvenile hormone (JH) by the corpora allata, the identity and role of the peptides involved is not clear. This paper describes in detail the neuroanatomy and role of three pairs of DH31-containing neurons and their essential role in inhibition under diapause-inducing conditions of JH synthesis by adult Drosophila female corpora allata. They show that these 3 pairs of cells (two lateral and one more central) correspond to the previously identified CA-LP1 and CA-LP2 cells in the larva and extend their axons to the corpora allata where they contact the gland cells. They find that small NPF Receptor is present in these DH31 cells which receive input from the nearby s-LNvs that contain sNPF and likely provide environmental input. After showing that reproductive dormancy was not achieved under diapause-inducing conditions in DH31 mutants, they also used a Gal4 driver specific for the DH31 neurons to both suppress and overexpress DH31 and determined the effects on ovarian maturation. In addition, they show that suppression of the DH31 receptor in the CA prevents the reproductive dormancy response by increasing the hemolymph titer of JH.

This is a well done, comprehensive study that clearly defines the essential role of the brain DH31 neurons in the inhibition of corpora allata function under diapause-inducing conditions. It is the first to show that a defined set of neurons control the production of JH in response to environmental conditions and thus mediate the adult diapause of Drosophila. The authors however should justify why they have decided to use “dormancy” rather than “diapause” in discussing this phenomenon as it seems an unnecessary change from accepted terminology in the insect literature.

Minor changes:
Line 132: cholecystokinin-8

Line 252: Clearer to say “…the axonal termini of s-LNvs were in close proximity to the neuronal…”

Lines 375-376: Reword these lines: for example, “….CA-LP neurons have a negative effect on JH biosynthesis during adult development such that male genital rotation occurs normally (Adam et al., 2003),...”

Line 408: In contrast…

Fig 2A: “glutamate” misspelled in label for DN1p neurons Fig S2D, E: Y-axis should be “Grasp-negative”

© 2023. Published by The Company of Biologists under the terms of the Creative Commons Attribution License (https://creativecommons.org/licenses/by/4.0/).

Reviewer 3

Advance summary and potential significance to field

Diapause is a fascinating biological phenomenon that we do not understand how it occurs both in molecular and cellular terms. Drosophila females undergo reproductive diapause (also called as reproductive dormancy) in response to a combination of short day lengths and low temperature conditions. Thus, juvenile hormones (JH), the major gonadotropins of this insect species, are very likely to be involved in this biological process. In this study, Kurogi et al. discovered a subset of Dh31 neurons (i.e., CA-LP) innervating corpora allata (CA), an endocrine organ that produces and secretes JH, and presented evidence that Dh31 expressed in CA-LP is involved in the oogenesis inhibition under reproductive dormancy-inducing conditions. In addition, the authors reported that the Dh31 receptor (Dh31R) is expressed in CA, along with the evidence that Dh31R-RNAi in CA alleviates the inhibition of JH production induced by reproductive dormancy. Based on these results, the authors made a claim that this Dh31 system is essential for female reproductive dormancy. Considering limits and caveats in their experimental results, however I feel that the authors need to tone down their claims.

Comments for the author

I hope the authors address following questions in the revised manuscript.

Major issues
1. Do Dh31 in CA-LP and Dh31R in CA affect oogenesis or vitellogenesis under control conditions that do not induce reproductive dormancy?
2. Does (forced) activation of CA-LP induce reproductive dormancy under control conditions?
3. Does the neural activity of CA-LP change in response to conditions that induce reproductive dormancy?
4. In Figure 3 and Figure 4, the scale (y-axis) changes even in the same figure because the intensity of the phenotype changes significantly depending on the experiment. The authors need to give reasons for this variability.
5. CA-LP seems to project to the brain PI area, where IPC is located. IPC has been associated with reproductive dormancy several times. Thus, the authors should test the possibility that Dh31 also regulates IPC. For example, Dh31R-RNAi in IPCs? Dh31R expression in IPCs?

Minor issues
1. Figure 2. Clock neurons that make synaptic contact with CA-LP2 make up no more than 5% of all synaptic connections. Thus, the authors should provide comprehensive summary of EM data without being limited to clock neurons. If possible, try to validate some of EM data using genetic tools, such as Trans-Tangol
2. Figure 2. To support the claim that CA-LP receives synaptic input from s-LNv expressing PDF and sNPF, the authors examined whether CA-LP expresses PDFR and sNPFR. However, in addition to s-LNv, there are many brain cells expressing PDF or sNPF, so this result is not meaningful.
3. Aug21-Gal4 is expressed in cells other than CA. In this study, the authors also used JHAMT-Gal4, more specific for CA than Aug21-Gal4. The authors should repeat at least a few major Dh31R-RNAi experiments with the JHAMT-Gal4 driver.
4. JH acts not only on fat bodies, but also on ovaries. Revise the Figure 6D.
5. Provide evidence for the efficacy of transgenic RNAis on Dh31R.
6. R94H10 and Dh31 expression pattern in the VNC in supplemental information

---

First revision

Author response to reviewers' comments

Reviewer 1:

However, crucial evidence is still missing to support the claim that the CA-LP2 neurons play an essential role in regulating temperature-dependent reproductive dormancy. In other words, the

© 2023. Published by The Company of Biologists under the terms of the Creative Commons Attribution License (https://creativecommons.org/licenses/by/4.0/).

context for Dh31 signaling to regulate female reproduction is unclear and the authors have yet to demonstrate environmental temperature is this context.
Response: Thank you very much for providing us with valuable feedback to improve our manuscript. We have carefully considered your criticisms and conducted multiple experiments to address most of the concerns raised in Reviewer 1's comments.

1. Although perturbing Dh31 signaling is able to change JH titer and ovary morphology under the extreme environmental condition, it is a different thing to say that the communication between the brain and the CA via Dh31 is the major pathway for environmental condition to change the reproductive status. The authors have not shown what has been changed by the extreme environmental condition.
Does environmental condition change the level of Dh31, Dh31R or the excitability of the CA-LP2 neurons? Looking at the functional role of the CA-LP2 neurons, Dh31 and Dh31R in females under normal environmental conditions (25 degree and 12:12 light condition) may provide an opportunity to compare and contrast in order to identify the factor(s) that change with the environmental conditions. For example, a recent published study (Meiselman et al, 2022) showed that AstC expression is temperature dependent. However, the AstC-expressing clock neurons DN3 do not seem to synapse with the CA-LP neurons (Figure 2). As such, it is possible that DN3 is part of a different pathway to regulate reproductive dormancy. It is therefore imperative for the authors to resolve these outstanding issues.
Response: Thank you for your insightful comments on the role of DH31 in regulating reproductive dormancy. We agree that these issues are important and have conducted four experiments to address your concerns. We have also included new data on L333-351 and L359-361 in the revised manuscript. Specifically, we have performed the following experiments:

1) **Detection of *Dh31-GAL4>UAS-GFP* signal in CA-LP neurons to examine the transcriptional regulation of *Dh31* (Fig. S4A).**
2) **Immunohistochemical experiment using anti-DH31 antibody to examine DH31 protein levels in CA-LP neurons (Fig. S4B).**
3) **Detection of *Dh31R-GAL4>UAS-GFP* signal in the CA to examine the transcriptional regulation of *Dh31R* (Fig. S5A). Unfortunately, we found that the anti-DH31R antibody (generated by Dr. Fumika Hamada's lab) did not work well for immunostaining the CA.**
4) **Detection of TRIC signals (a transcriptional reporter of intracellular Ca^{2+}) in CA-LP neurons (Fig. S4C,D). TRIC is a technique to quantify neuronal activity.**

From these analyses, we found that Dh31 expression levels were upregulated in CA-LP1 neurons but remained unchanged in CA-LP2 neurons under dormancy-inducing conditions (Fig. S4A). In contrast, DH31 protein levels were notably reduced in both CA-LP1 and CA-LP2 neurons under dormancy-inducing conditions (Fig. S4B). These results might reflect enhancement of Dh31 transcription, at least in CA-LP1 neurons, and DH31 release from CA-LP neurons in response to dormancy- inducing conditions. However, we did not observe any apparent differences in TRIC signals in CA-LP neurons or *Dh31-R* expression levels in the CA between dormancy- and non-dormancy-inducing conditions.
Therefore, although we have demonstrated that CA-LP neurons are regulated in response to dormancy-inducing, the exact mechanism of this regulation is still unclear. In the revised manuscript, we have faithfully discussed these points (L493-502). We hope that the reviewer would find the manuscript revised appropriately.

2. Related to the above concern, the authors need to be forthcoming with the conditions for the imaging experiments presented in Figure 5. Lines 591-592 in Methods indicate that for these imaging experiments, CA were obtained from newly eclosed virgin females. These are not females under low-temperature and short light exposure. Therefore, it is possible that Dh31 would not trigger cAMP increases in females under reproductive dormancy. Furthermore, collagenase treatment can also dissolve synaptic connections. A more physiologically relevant experiment is to stimulate the CA-LP2 neurons, perhaps with optogenetics or the ATP-P2X2 system, while imaging the CA. With this experimental regimen, one can determine whether Dh31 signaling is required for the change of intracellular cAMP and whether CA-LP2 is the major conduit linking environmental conditions to CA function.
Response: We appreciate Reviewer 1's constructive criticism and suggestions. We have addressed their comments by conducting additional experiments and including the results in

© 2023. Published by The Company of Biologists under the terms of the Creative Commons Attribution License (https://creativecommons.org/licenses/by/4.0/).

the revised manuscript. Specifically:

1) **We conducted cAMP imaging of the CA dissected from dormant flies and found that the dormant flies' CA also responded to DH31 peptide in our *ex vivo* culture system. We have included these data in the Results section (L412-413) and in Fig. 5D and 5E of the revised manuscript.**
2) **We employed the ATP-P2X2 system to stimulate CA-LP neurons *ex vivo,* as suggested by the reviewer. Although we observed a trend of increase in intracellular cAMP levels in the CA after ATP administration, the difference between the groups did not reach statistical significance. We believe that the preparation of the brain-CA complex was technically challenging, and we may have underestimated the actual effect of the synaptic DH31 transmission to the CA. We have decided to include these data and our hypothesis in the Results section (L417-434) and Fig. 5H, 5I, and S6E of the revised manuscript.**

Of note, unfortunately, the optical stimulation system using CsChrimson cannot be used in our study because the excitation wavelength required to activate CsChrimson significantly overlaps with the excitation wavelength of PinkFlamindo, which we use to monitor cAMP levels in the CA. This means that we cannot monitor cAMP levels without CsChrimson activation.

3. It is counterintuitive to think that an increase of intracellular cAMP concentration would reduce juvenile hormone release. The authors would need to demonstrate this empirically, perhaps by perturbing adenylyl cyclase or phosphodiesterase in the AC.

Response: We would like to remind the reviewer that the negative impact of intracellular cAMP on JH biosynthesis has been previously reported by Andreatta et al. in Scientific Reports 2018, which we have cited in the manuscript. The paper showed that the dominant-negative form of cAMP- dependent protein kinase (Protein Kinase A, PKA) enhances JH biosynthesis.

In response to the reviewer's suggestion, we examined the reproductive dormancy phenotype in *JHAMT-GAL4>dominant-negative PKA* (referred to as *PKA[DN]*) under dormancy-inducing conditions. We found that these flies produced more mature eggs than control flies under dormancy-inducing conditions. This phenotype is consistent with the positive impact of PKA[DN] on JH biosynthesis reported by Andreatta et al. We have included these findings in the Results section (L435-445) and in Fig. S7A and B of the revised manuscript.

4. Figure 3. According to Janelia Flylight, R94H10 is also expressed in VNC, not just CA-LP. The authors should perform experiments to assess whether VNC neurons are relevant.

Response: We followed the suggestion of Reviewer 1 and studied transgenic flies carrying both *R94H10-GAL4* and *tsh-GAL80,* which suppress GAL4 activity in the VNC. We found that these animals still exhibited a higher level of mature egg production even under dormancy- inducing conditions, suggesting that DH31 in the VNC is not involved in regulating reproductive dormancy. We have described these data in the Results section (L301-312) and presented them in Fig. 3G-I of the revised manuscript. We have also included the expression pattern of *R94H10-GAL4* and *R94H10-GAL4 tsh-GAL80* lines in Fig. S1J, S3C, and S3D.

Minor concerns:

1. The upstream regulators of CA-LP are not well characterized. Due to the insufficient evidence to support the notion that the clock neurons are functionally upstream of CA- LP, I would suggest this figure to be a supplementary figure. Specifically, the indicated neurons as well as the neuropeptides or neurotransmitters in Fig 2A are not experimentally validated to be upstream of CA-LP2. A counter example is provided by the authors that the PDF receptor is not expressed in CA-LP. Fig 2A gives the impression that all indicated neuropeptides and neurotransmitters are utilized to regulate CA-LP2. This may be misleading the readers. Furthermore, it is unclear whether sNPF receptor is relevant to the dormancy phenotype. To support this, it will be necessary to perform genetic perturbation experiments, such as knocking down sNPF receptor in CA-LP, to demonstrate that clock neurons are upstream regulators of CA-LP.

Response: We understand Reviewer 1's criticisms. We agree that exploring the role of the CA-LP upstream neurons in reproductive dormancy is a useful topic to investigate in future work. However, we believe that this is beyond the scope of our current study.

© 2023. Published by The Company of Biologists under the terms of the Creative Commons Attribution License (https://creativecommons.org/licenses/by/4.0/).

In this regard, we have followed the reviewer's suggestion, and moved all the items of the original Figure 2 to a supplemental figure (Fig. S2). On the other hand, we still believe that the connectome data are valuable because of the consideration of the similarity of the dormancy- inducing neurons in other insects.

Reviewer 2:
The authors however should justify why they have decided to use "dormancy" rather than "diapause" in discussing this phenomenon as it seems an unnecessary change from accepted terminology in the insect literature.
Response: Thank you very much for raising this important point. In our recent review paper [Kurogi et al. (2021)], we discussed the definition of the term 'dormancy.' In general, dormancy can be broadly classified into two types: diapause and quiescence. Diapause is induced by seasonal cues that do not directly prevent development, but rather predicts the arrival of unfavorable conditions. On the other hand, quiescence is characterized by slower metabolism and directly results from unfavorable environmental conditions such as low temperature. There is still debate about whether the suppression of oogenesis in winter-season conditions in *D. melanogaster* should be classified as diapause or quiescence. For these reasons, most papers published in recent years simply use the term "dormancy" to describe the short-day and low-temperature-induced changes in *D. melanogaster* adults. Therefore, in this paper, we used the term 'reproductive dormancy' to refer to the suppression of oogenesis under diapause-inducing conditions, in accordance with recent studies.

In the revised manuscript, we have briefly clarified these points by adding the following sentences to the fourth paragraph of the Introduction. "In most recent pieces of literature, the reproductive diapause-like status of *D. melanogaster* is frequently called 'reproductive dormancy' (See Kurogi et al., 2021 for detailed discussion). Therefore, in accordance with recent studies, hereafter, we will use the term 'reproductive dormancy' to refer to suppressing oogenesis under diapause-inducing conditions" (L136-139). Additionally, in the Abstract and the first and second paragraphs of the Introduction, we have replaced 'dormancy' with 'diapause' as we describe the phenomenon of diapause in general. We hope that the revised manuscript will be acceptable to the reviewer.

Minor changes:
Line 132: cholecystokinin-8
Response: We have fixed this error (L127).

Line 252: Clearer to say "…the axonal termini of s-LNvs were in close proximity to the neuronal…"
Response: We have fixed this error (L255-256).

Lines 375-376: Reword these lines: for example, "….CA-LP neurons have a negative effect on JH biosynthesis during adult development such that male genital rotation occurs normally (Adam et al., 2003),..."
Response: To clarify the meaning of this sentence, we have rewritten the sentence as follows: "Previous studies have indicated that CA-LP neurons have a negative effect on JH biosynthesis during pupal development, permitting a normal male genital rotation…" (L482-485).

Line 408: In contrast…
Response: We have fixed this error (L520).

Fig 2A: "glutamate" misspelled in label for DN1p neurons
Response: We have fixed this error in Fig. S2E in the revised manuscript.

Fig S2D, E: Y-axis should be "Grasp-negative"
Response: We have fixed this error in Fig. 2C and 2D in the revised manuscript.

Reviewer 3:
Major issues
1. Do Dh31 in CA-LP and Dh31R in CA affect oogenesis or vitellogenesis under control conditions that do not induce reproductive dormancy?

© 2023. Published by The Company of Biologists under the terms of the Creative Commons Attribution License (https://creativecommons.org/licenses/by/4.0/).

Response: We conducted RNAi experiments to specifically knock down *Dh31* in CA-LP, as well as knock down the *Dh31R* specifically in the CA under non-dormancy-inducing conditions. We then counted the number of mature eggs produced by these flies. Our results showed no difference in mature egg production between control and loss-of-*Dh31* function females or loss-of-*Dh31-R* function females under non-dormancy-inducing conditions. These results suggest that DH31-DH31-R signaling is specifically required for suppressing oogenesis under dormancy-inducing conditions, but not under non-dormancy-inducing conditions. We have described these data in the Results section (L294-300 and L376-386) and in Fig. S3A, S3B, S5G-I of the revised manuscript.

2. *Does (forced) activation of CA-LP induce reproductive dormancy under control conditions?*
Response: To address this question, we conducted a new experiment in which the *TrpA1* channel gene was forcibly expressed specifically in CA- LP to activate the neurons under normal conditions, and then counted the number of mature eggs. We found that at the restrictive temperature (29°C), the *TrpA1*-overexpressing virgin females had fewer mature eggs compared to controls at the permissive temperature (Fig. S3E), suggesting that CA-LP neurons are sufficient to suppress oogenesis. We have described these data in the Results section (L320-328) and in Fig. S3E.

3. *Does the neural activity of CA-LP change in response to conditions that induce reproductive dormancy?*
Response: We agree with the reviewer that understanding the role of DH31 in regulating reproductive dormancy is important. To address this question, we used the TRIC technique to evaluate the signals of intracellular Ca^{2+} in CA-LP neurons, which can reveal differences in neuronal activities between normal and dormancy-inducing conditions. However, we did not observe any apparent differences in TRIC signals in CA-LP neurons between dormancy- and non-dormancy-inducing conditions. Therefore, we currently cannot say whether and how the neuronal activity of CA-LP neurons is regulated in response to dormancy- inducing conditions. We have described these data in the Results section (L344-351) and in Fig. S4C and S4D, and we have also discussed these issues in the manuscript (L493-502). We hope that the revised manuscript addresses the reviewer's concerns appropriately.

4. *In Figure 3 and Figure 4, the scale (y-axis) changes even in the same figure because the intensity of the phenotype changes significantly depending on the experiment. The authors need to give reasons for this variability.*
Response: With respect to the phenotype of mature egg production phenotype in this study, we have not figured out the exact reason for the variability that Reviewer 3 pointed out. It is possible that an anonymous nucleotide polymorphism may lead to different outcomes of mature egg numbers. Elucidating such genetic variances to affect reproductive dormancy might also be an interesting issue for future study. We appreciate the reviewer's important comment.

5. *CA-LP seems to project to the brain PI area, where IPC is located. IPC has been associated with reproductive dormancy several times. Thus, the authors should test the possibility that Dh31 also regulates IPC. For example, Dh31R-RNAi in IPCs? Dh31R expression in IPCs?*
Responses: First and foremost, we want to emphasize that the axon termini of CA-LP neurons, which were marked by Syt-GFP, were not observed in the PI area (Fig. 2A). Therefore, we respectfully disagree with the reviewer's opinion that CA-LP neurons project to the PI.
Regarding the reviewer's suggestion, we conducted additional experiments to investigate the role of DH31-R in the IPCs. We confirmed that the *DH31-R-t2A-GAL4* driver was inactive in the IPCs marked with anti-DILP2 antibody. Furthermore, *Dilp-2-GAL4*-driven *Dh31-R* RNAi did not enhance mature egg productions compared with control under dormancy-inducing conditions. These findings suggest that DH31-R acts in the CA rather than the IPCs to regulate reproductive dormancy. We have included these results in the Results section (L376-386) and in Fig. S5D-F of the revised manuscript.

Minor issues
1. *Figure 2. Clock neurons that make synaptic contact with CA-LP2 make up no more than 5%*

© 2023. Published by The Company of Biologists under the terms of the Creative Commons Attribution License (https://creativecommons.org/licenses/by/4.0/).

of all synaptic connections. Thus, the authors should provide comprehensive summary of EM data without being limited to clock neurons. If possible, try to validate some of EM data using genetic tools, such as Trans-Tangol.
Response: We would like to mention that the comprehensive EM dataset has already been provided in the supplemental dataset (S2). Based on these data, anyone can calculate the percentage of synaptic connections between CA-LP2 and any candidate neuron.
We agree that exploring the role of clock neurons upstream of CA- LP in reproductive dormancy is an important topic to investigate in future studies. However, this is beyond the scope of our current study. On the other hand, we still believe that the data showing the connection between CA-LP2 and clock neurons are valuable due to the consideration of the similarity of the dormancy-inducing neurons in other insects.
In the revised manuscript, we have followed Reviewer 1's suggestion and moved all the items of the original Fig. 2 (related to EM data) to a supplemental figure (Fig. S2).

2. *Figure 2. To support the claim that CA-LP receives synaptic input from s-LNv expressing PDF and sNPF, the authors examined whether CA-LP expresses PDFR and sNPFR. However, in addition to s-LNv, there are many brain cells expressing PDF or sNPF, so this result is not meaningful.*
Response: We appreciate Reviewer 3's opinion, but we only intended to provide a limited characterization of the CA-LP neurons in this study, and a more comprehensive analysis would require a substantial amount of time that is beyond the scope of this revision. However, if the reviewer and/or the Editor prefer that we remove the PDF and sNPF data (in Fig. S2H and S2I), we can do so. Nevertheless, we still believe that these data may be useful to readers who are interested in insect reproductive dormancy, and we are happy to share them.

3. *Aug21-Gal4 is expressed in cells other than CA. In this study, the authors also used JHAMT-Gal4, more specific for CA than Aug21-Gal4. The authors should repeat at least a few major Dh31R-RNAi experiments with the JHAMT-Gal4 driver.*
Response: To address this point, we conducted a new experiment to examine mature egg formation in *JHAMT-GAL4>UAS-Dh31-R-RNAi* animals. We confirmed that these animals also exhibited similar reproductive phenotypes as *AUG21-GAL4>UAS-Dh31-R-RNAi* animals. We have added and presented these data in the main text (L372-373) and in Fig. S5C of the revised manuscript.

Unfortunately, we are currently unable to conduct new JH titer measurements with *JHAMT-GAL4>UAS-Dh31R-RNAi* animals, as the authors (M.N. and F.G.N.), who measured JH titer in this study, have moved from Florida to the Czech Republic and are still in the process of setting up their protocols in the MS core facility. We hope the reviewer will understand the situation.

4. *JH acts not only on fat bodies, but also on ovaries. Revise the Figure 6D.* **Response: We have fixed this problem by adding the additional arrow from the CA to the ovary.**

5. *Provide evidence for the efficacy of transgenic RNAis on Dh31R.*
Response: To address this point, we performed RT-qPCR experiments to assess *Dh31R* expression levels in the whole body of *Actin-GAL4>UAS- Dh31R-RNAi* flies. We observed that forced expression of two independent Dh31-R IR constructs led to around 60% and 80% reduction of *Dh31-R* mRNA levels, respectively. We have included these new findings in the main text (L365-367) and Fig. S5B of the revised manuscript.

We must acknowledge that it is technically demanding to measure *Dh31R* expression levels exclusively in the CA by RT-qPCR since obtaining the small CA without any cross-contamination from other tissues is unfeasible for us. Moreover, we discovered that the anti-DH31R antibody (generated by Dr. Fumika Hamada's lab) did not yield satisfactory results for immunostaining the CA (our unpublished observation).

6. *R94H10 and Dh31 expression pattern in the VNC in supplemental information.*
Response: We have included the requested data in Fig. S1J of the revised manuscript.

© 2023. Published by The Company of Biologists under the terms of the Creative Commons Attribution License (https://creativecommons.org/licenses/by/4.0/).

Second decision letter

MS ID#: DEVELOP/2022/201186

MS TITLE: Female reproductive dormancy in*Drosophila*is regulated by DH31-producing neurons projecting into the *corpus allatum*

AUTHORS: Yoshitomo Kurogi, Eisuke Imura, Yosuke Mizuno, Ryo Hoshino, Marcela Nouzova, Shigeru Matsuyama, Akira Mizoguchi, Shu Kondo, Hiromu Tanimoto, Fernando G Noriega, and Ryusuke Niwa

I have now received all the referees reports on the above manuscript, and have reached a decision. The referees' comments are appended below, or you can access them online: please go to BenchPress and click on the 'Manuscripts with Decisions' queue in the Author Area.

The overall evaluation is positive and we would like to publish a revised manuscript in Development. Although no further experiments are strictly necessary, I fully agree with reviewer #1. I will leave it up to you to decide whether you want to address their point by conducting the suggested experiment, or by further discussing this issue more directly in your discussion, acknowledging the fact that in its absence, the model remains to be fully demonstrated.

Please also attend to all of the reviewers' comments in your revised manuscript and detail them in your point-by-point response. If you do not agree with any of their criticisms or suggestions explain clearly why this is so.

Reviewer 1

Advance summary and potential significance to field

The study by Kurogi et al addressed an important question of how unfavorable environmental conditions regulate female reproduction. It has been well established that Drosophila females will enter a state of reproductive dormancy in the winter when environmental temperature is lower and the daily period of light is shortened.
Furthermore, reproductive dormancy in insects is associated with a lower titer of juvenile hormone (JH) released from the corpora allata (CA). Here, the authors claimed that they have found the elusive brain-to-CA connection that regulates female reproductive dormancy through controlling JH.

Comments for the author

In the revised manuscript, the authors have taken several important steps towards establishing a mechanism for Dh31 signaling to regulate reproductive dormancy.
Specifically, what changes in the Dh31 signaling pathway cause an increase in CA cAMP concentration. The new experiments show that a dormancy condition increases Dh31 transcript in, but does not change the neural activity of, CA-LP neurons (Fig. S4). Together with their finding that thermogenetic activation of CA-LP neurons is sufficient to suppress oogenesis (Fig. S3), one would hypothesize that the increased expression of Dh31 in CA-LP neurons causes the elevation of cAMP in the CA. It is a pity that the authors did not test whether overexpression of Dh31 in the CA-LP neurons is sufficient to induce reproductive dormancy. To corroborate this test, the authors could expand the P2X2 experiments in Fig. 5H in an effort to determine whether the change of Dh31 transcription results in differential cAMP responses in the CA of dormant and normal females. Technically it is possible to accommodate cAMP imaging and optogenetic stimulation of CA-LP neurons in the same prep by interleaving the two light stimulations [my response to the authors' response to my previous Pt.2].
I should emphasize here that the authors have made significant progress in the revised manuscript. My comments should be viewed as constructive criticism rather than a barrier to publication. I would encourage the senior author to address this interesting and important biological problem to the best of their ability without leaving unsolved issues to the next paper. The issue here is, simply put whether a change in Dh31 signaling underlies reproductive dormancy. In my opinion, the case is weaker than what the authors can build in a reasonable amount of time.

© 2023. Published by The Company of Biologists under the terms of the Creative Commons Attribution License (https://creativecommons.org/licenses/by/4.0/).

Reviewer 2

Advance summary and potential significance to field

This paper is the first to show that a defined set of neurons control the production of juvenile hormone in response to environmental conditions and thus mediate the adult diapause of Drosophila. This paper provides a firm basis for further elucidation of how environmental signals can disrupt the normal endocrine control of egg maturation. It also should stimulate the search in other species for a similar role for the same peptide or other peptides with the same function.

Comments for the author

This revised version is much improved and ready for publication.
One minor correction is needed in Figure 2C and D. The y axes in both should be "GRASP-negative"

Reviewer 3

Advance summary and potential significance to field

I found that the revised manuscript successfully addressed all of my concerns and comments.

Comments for the author

Now I have a few minor comments regarding for the revised portion of the manuscript.

On line 336, I suggest clarifying in the main text that Dh31 expression is measured by quantifying Dh31-Gal4 activity.

Fig S4B, which provide evidence of Dh31 secretion from CA-LP neurons, is important and should be presented in a main figure along with representative confocal images.

On line 422, the statement “and placed in a glass-bottomed dish filled with culture medium” is too trivial to be included in the Results section and should be moved to the Methods section.

On line 431, it is not necessary to include the statement “speculate that we may have underestimated the actual effect of the synaptic DH31 transmission to the CA”

Second revision

Author response to reviewers' comments

Reviewer 1:

The study by Kurogi et al addressed an important question of how unfavorable environmental conditions regulate female reproduction. It has been well established that Drosophila females will enter a state of reproductive dormancy in the winter when environmental temperature is lower and the daily period of light is shortened.
Furthermore, reproductive dormancy in insects is associated with a lower titer of juvenile hormone (JH) released from the corpora allata (CA). Here, the authors claimed that they have found the elusive brain-to-CA connection that regulates female reproductive dormancy through controlling JH.

In the revised manuscript, the authors have taken several important steps towards establishing a mechanism for Dh31 signaling to regulate reproductive dormancy. Specifically, what changes in the Dh31 signaling pathway cause an increase in CA cAMP concentration. The new experiments show that a dormancy condition increases Dh31 transcript in, but does not change the neural activity of, CA-LP neurons (Fig. S4). Together with their finding that thermogenetic activation of CA-LP neurons is sufficient to suppress oogenesis (Fig. S3), one

© 2023. Published by The Company of Biologists under the terms of the Creative Commons Attribution License (https://creativecommons.org/licenses/by/4.0/).

would hypothesize that the increased expression of Dh31 in CA-LP neurons causes the elevation of cAMP in the CA. It is a pity that the authors did not test whether overexpression of Dh31 in the CA-LP neurons is sufficient to induce reproductive dormancy. To corroborate this test, the authors could expand the P2X2 experiments in Fig. 5H in an effort to determine whether the change of Dh31 transcription results in differential cAMP responses in the CA of dormant and normal females. Technically, it is possible to accommodate cAMP imaging and optogenetic stimulation of CA-LP neurons in the same prep by interleaving the two light stimulations [my response to the authors' response to my previous Pt.2].

I should emphasize here that the authors have made significant progress in the revised manuscript. My comments should be viewed as constructive criticism rather than a barrier to publication. I would encourage the senior author to address this interesting and important biological problem to the best of their ability without leaving unsolved issues to the next paper. The issue here is, simply put, whether a change in Dh31 signaling underlies reproductive dormancy. In my opinion, the case is weaker than what the authors can build in a reasonable amount of time.

Response: We deeply appreciate the reviewer's critical comments and suggestions. We fully agree with the reviewer that our current data are insufficient to answer the reviewer's question of whether the change of Dh31 transcription (as well as DH31 protein levels) results in differential cAMP responses in the CA of dormant and normal females. As the reviewer suggested, we do not want to leave this unsolved issue either. However, considering realistic timelines (particularly for the first author with little time remaining before his Ph. D. course graduation), it is very difficult for us to additionally conduct the number of important experiments that the reviewer thoughtfully pointed out. For the second round of revision, the Editor stated that she leaves it up to us to decide whether we address the reviewer's point by conducting the suggested experiment or by further discussing this issue more directly. In conclusion, in the revised manuscript, we have not included the suggested new experiments, but stated in the Discussion section that the model we proposed remains to be fully demonstrated (L502-510).

Meanwhile, we have also decided to include a new experimental dataset, which is not a direct answer to the reviewer, but provides additional insights into the role of DH31. As the reviewer suggested, we have expanded the P2X2 experiments to determine if P2X2-stimulation changes DH31 levels in CA-LP neurons. We found that DH31 protein levels in CA-LP neurons were reduced in the ATP-treated brain-CA complexes when compared to the vehicle-treated ones. This significant observation is consistent with our data showing that DH31 protein levels in CA-LP neurons were reduced in dormancy-inducing conditions (Fig. 3M, N). Therefore, the new results further support the idea that the neuronal regulation of DH31 release might be essential for regulating reproductive dormancy. We have included these new data in the Results section (L432-436) and in the main Fig. 5J and K. In this regard, we have also added a brief sentence to describe this experiment in the Materials and Methods section (L770-772).

Reviewer 2:

This paper is the first to show that a defined set of neurons control the production of juvenile hormone in response to environmental conditions and thus mediate the adult diapause of Drosophila. This paper provides a firm basis for further elucidation of how environmental signals can disrupt the normal endocrine control of egg maturation. It also should stimulate the search in other species for a similar role for the same peptide or other peptides with the same function.

Response: We are glad to hear this positive comment.

This revised version is much improved and ready for publication.
One minor correction is needed in Figure 2C and D. The y axes in both should be "GRASP-negative"

Response: We have fixed the error as indicated.

© 2023. Published by The Company of Biologists under the terms of the Creative Commons Attribution License (https://creativecommons.org/licenses/by/4.0/).

Reviewer 3:

I found that the revised manuscript successfully addressed all of my concerns and comments.
Response: We are glad to hear this positive comment.

Now I have a few minor comments regarding for the revised portion of the manuscript. On line 336, I suggest clarifying in the main text that Dh31 expression is measured by quantifying Dh31-Gal4 activity.
Response: As the reviewer suggested, we have rewritten the sentences (L338-340).

Fig S4B, which provide evidence of Dh31 secretion from CA-LP neurons, is important and should be presented in a main figure along with representative confocal images.
Response: We have followed the reviewer's suggestion and moved the original Fig. S4B to the main Fig. 3N. In addition, we have also included the representative image requested in the main Fig. 3M.

On line 422, the statement "and placed in a glass-bottomed dish filled with culture medium" is too trivial to be included in the Results section and should be moved to the Methods section.
Response: As the reviewer suggested, we have deleted the sentence from the Results section (L425 in the revised manuscript). In the Materials and Methods section, we have already provided the detailed protocol (L743- 746).

On line 431, it is not necessary to include the statement "speculate that we may have underestimated the actual effect of the synaptic DH31 transmission to the CA"
Response: We have deleted the sentence from the Results section as suggested (L436 in the revised manuscript).

Third decision letter

MS ID#: DEVELOP/2022/201186

MS TITLE: Female reproductive dormancy in *Drosophila* is regulated by DH31-producing neurons projecting into the *corpus allatum*

AUTHORS: Yoshitomo Kurogi, Eisuke Imura, Yosuke Mizuno, Ryo Hoshino, Marcela Nouzova, Shigeru Matsuyama, Akira Mizoguchi, Shu Kondo, Hiromu Tanimoto, Fernando G Noriega, and Ryusuke Niwa
ARTICLE TYPE: Research Article

I am happy to tell you that your manuscript has been accepted for publication in Development, pending our standard ethics checks.

© 2023. Published by The Company of Biologists under the terms of the Creative Commons Attribution License (https://creativecommons.org/licenses/by/4.0/).
